# Global burden of cardiovascular diseases attributable to diet low in vegetables from 1990 to 2021 and forecasting the future trends: a population-based study

**DOI:** 10.3389/fcvm.2024.1491869

**Published:** 2025-01-15

**Authors:** Qingsong Mao, Yuzhe Kong

**Affiliations:** ^1^ Hepatobiliary Pancreatic Surgery, Banan Hospital Affiliated of Chongqing Medical University, Chongqing, China; ^2^ Xiangya School of Medicine, Central South University, Changsha, China

**Keywords:** cardiovascular diseases, vegetables, mortality forecasting, epidemiology, disease burden

## Abstract

**Background:**

This investigation examines the worldwide impact of cardiovascular diseases (CVD) resulting from inadequate vegetable consumption, based on the 2021 Global Burden of Disease Study data.

**Method:**

The study assessed the global, regional, and national repercussions of low vegetable intake on CVD, with a focus on variations among different age and gender demographics. It further analyzed the correlation between disease burden and the Socio-Demographic Index (SDI), and employed an ARIMA model to predict future trends in CVD associated with insufficient vegetable consumption up to 2050.

**Result:**

In 2021, a diet lacking in vegetables was responsible for roughly 682,400 deaths and 16 million disability-adjusted life years (DALYs) attributed to CVD, indicating a declining pattern over recent year. Individuals aged 75 and older were predominantly affected. Future projections indicate an expected rise in CVD incidence in lower-middle SDI regions, with African nations potentially experiencing increased challenges related to low vegetable consumption by 2030 and 2050.

**Conclusion:**

The findings underscore the critical necessity for preventive measures against CVD and emphasize the significance of improving dietary habits, particularly vegetable intake.

## 1 Introduction

Cardiovascular diseases (CVD) represent the foremost cause of mortality and morbidity globally, encompassing a diverse array of disorders affecting the heart and blood vessels, including coronary artery disease, stroke, and heart failure. In 2019, CVD affected an estimated 523 million individuals worldwide and was responsible for 18.6 million deaths (1, 2). The widespread prevalence of CVD imposes significant economic and healthcare burdens, particularly in low and middle-income countries (2). Given the enormity of its impact, there is a critical need to explore modifiable risk factors that contribute to the prevention and management of CVD.

Recent emphasis on dietary habits, particularly the intake of fruits and vegetables, highlights a significant, modifiable dietary factor. Fruits and vegetables are not only cost-effective and accessible but also provide substantial health benefits that may reduce the risk of chronic diseases such as CVD and diabetes (3–16). Public health authorities and national dietary guidelines consistently advocate for increased consumption of these food groups, aligning with evidence suggesting their role in enhancing cardiovascular health.

However, the specific impact of fruit and vegetable consumption on different types of CVD is less frequently dissected. A nuanced understanding is necessary, as certain cardiovascular conditions may be more sensitive to dietary modifications than others. Furthermore, a host of socioeconomic, environmental, and behavioral factors influence dietary habits. These include the availability and affordability of fruits and vegetables, cultural dietary patterns, and regional disparities in health education and awareness. For instance, variations in fruit and vegetable consumption can be attributed to economic accessibility, with individuals in lower socioeconomic strata or remote areas facing significant challenges in obtaining fresh produce (17).

Additionally, lifestyle choices accumulated over decades, especially in individuals aged 75 and above, profoundly affect CVD risks. This demographic's dietary patterns earlier in life, along with their physical activity levels, play a crucial role in their current health status. While our study focuses on the effects of insufficient vegetable consumption on CVD, a comprehensive analysis would also consider these broader lifestyle factors to paint a fuller picture of CVD dynamics (18).

By addressing these elements, this research aims to provide a holistic view of the role of diet in cardiovascular health and to underscore the potential of dietary interventions in reducing the global burden of CVD.

## 2 Method

### 2.1 Study population

This research employed data from the 2021 Global Burden of Disease Study, reviewing 369 diseases and injuries and 87 risk factors across 204 nations from 1990 to 2021 (19).

To determine the impact of cardiovascular diseases (CVD), we utilized established methodologies from prior studies (20). We refined raw data sourced from health surveys and verbal autopsies to increase precision (21). This data was processed using the Cause of Death Ensemble model (CODEm), generating annual, region-specific CVD mortality estimates by age and gender (20, 22). We performed a comparative risk assessment to pinpoint critical CVD risk factors and computed population attributable fractions (PAF) to evaluate the impact of insufficient vegetable intake on CVD incidence. These PAFs were instrumental in estimating CVD-related mortality and disability-adjusted life years (DALYs) across varying demographics and periods (20). DALYs were calculated by adding years lost due to premature death from lower respiratory infections (LRIs) to years lived with disability (YLDs), with adjustments made for the severity of the condition (21). The Socio-Demographic Index (SDI) was developed considering elements such as fertility rates for those under 25 years (TFU25), educational attainment for individuals over 15 years (EDU15+), and per capita income, categorizing 204 locales into five SDI brackets (21, 23).

Definition of vegetable intake can be found in https://ghdx.healthdata.org/gbd-2021.

### 2.2 Statical analysis

We standardized mortality and DALY figures across different demographic structures using age-adjusted rates (AAR). Linear regression was performed on the natural logarithms of these metrics, formulated as y = *α* + *β*x + *ε*, where x denotes the year, and y is the natural log of the rate. The estimated annual percentage change (EAPC) was derived using the expression 100 * (e^*β*−1), accompanied by a 95% confidence interval (95% CI). An increase in AAR was noted if both the EAPC and the lower limit of the 95% CI were positive, a decline was observed if both the EAPC and the upper limit of the 95% CI were negative, and stability was concluded if neither condition was met (24, 25). The correlation between AAR and SDI was explored using Gaussian process regression with Loess smoothing, and Spearman rank correlation tests were utilized to examine this relationship (23, 25, 26).

Furthermore, an ARIMA (Autoregressive Integrated Moving Average) model was utilized to examine and forecast the influence of low vegetable intake on CVD trends on a global, regional, and national scale from 2020 to 2050. The configuration of the ARIMA model (p, d, q) included “p” as the number of lag observations for the autoregressive component, “q” for the lag of forecast errors in the moving average segment, and “d” for the differencing needed to stabilize the data. The selection of the model was guided by the Akaike Information Criterion (AIC) and the Bayesian Information Criterion (BIC) (27).

Data analysis was done using R. *P* < 0.05 was considered statistically significant (28–33).

## 3 Result

### 3.1 Spatiotemporal patterns of CVD attributable to diet low in vegetables

In 2021, insufficient intake of vegetables was associated with around 682,400 deaths and 16 million disability-adjusted life years (DALYs) due to cardiovascular diseases (CVDs). The age-standardized mortality rate (ASMR) stood at 8.2154 (95% UI, 5.6150–10.8400), and the age-standardized DALY rate (ASDR) was 187.4715 (95% UI, 122.4929–247.5979) per 100,000 individuals. There has been a notable reduction in the global impact of CVDs related to low vegetable consumption over the last thirty years (Table 1, Supplementary Table S1).

**Table 1:** Global and regional deaths and DALYs of CVD attributable to diet Low in vegetables in 1990 and 2021 in 27 global regions.

Location	Deaths Number in 1990	Deaths Number in 2021	ASMR in 2021	DALY Number in 1990	DALY Number in 2021	ASDR in 2021
Global	557,760.8414 (742,379.0859, 367,140.8153)	682,398.9340 (900,294.8892, 465,824.2366)	8.2154 (10.8400, 5.6150)	14,114,700.9788 (18,941,602.5246, 9,024,860.1820)	16,001,496.5356 (21,138,422.4135, 10,422,322.4342)	187.4715 (247.5979, 122.4929)
Region						
East Asia	126,792.3926 (165,431.1985, 90,492.7190)	38,215.7738 (66,626.6339, 17,630.6815)	2.2698 (3.9694, 1.0282)	2,929,449.0096 (3,851,010.1466, 2,115,439.6546)	600,527.6777 (1,041,523.2468, 291,211.2714)	32.5523 (56.3217, 15.9226)
Southeast Asia	65,422.0858 (91,989.9455, 35,387.2891)	88,467.9347 (117,378.1493, 57,156.2830)	14.9044 (19.8190, 9.8166)	1,934,439.1292 (2,762,803.8004, 958,495.6252)	2,375,595.6386 (3,153,702.0349, 1,520,344.5781)	351.1880 (464.1442, 225.9775)
Oceania	878.4122 (1,240.6363, 449.8130)	1,797.2468 (2,533.0139, 980.0237)	26.0572 (36.0129, 14.6517)	27,608.4413 (39,087.9642, 13,681.4482)	56,400.7429 (80,495.8499, 29,858.0364)	652.5225 (920.6227, 357.5523)
Central Asia	5,014.7781 (7,489.2004, 2,819.2767)	2,889.2894 (4,413.9938, 1,736.8818)	4.6724 (7.1090, 2.8257)	119,811.0049 (178,690.0204, 68,245.7052)	55,294.3762 (86,442.6182, 32,517.5129)	76.7550 (116.4924, 45.5306)
Central Europe	15,542.2260 (22,828.4511, 9,439.4440)	13,472.5134 (19,194.2598, 8,675.1027)	5.6655 (8.0934, 3.6439)	334,322.7071 (492,705.7827, 203,967.7594)	209,808.1958 (302,200.4026, 131,230.0348)	93.7168 (135.2973, 58.2624)
Eastern Europe	25,819.9152 (39,633.9334, 13,248.2653)	29,155.8055 (44,395.2969, 15,971.7245)	8.2450 (12.5461, 4.5172)	593,145.4537 (894,526.4277, 316,408.7481)	594,134.8289 (907,561.3560, 326,385.7864)	175.1513 (264.8592, 96.8721)
High-income Asia Pacific	5,785.7553 (8,301.4868, 3,739.0232)	8,207.5755 (12,260.7802, 4,793.7439)	1.1789 (1.7464, 0.7144)	112,178.2929 (160,341.5969, 71,937.1047)	109,180.8214 (161,876.5477, 67,317.6424)	21.1314 (31.2384, 13.1608)
Australasia	1,660.6883 (2,464.4090, 865.6780)	1,477.1402 (2,107.1792, 886.0258)	2.4163 (3.4252, 1.4524)	34,182.5591 (49,979.4332, 18,371.8282)	24,409.4327 (34,163.5476, 15,217.2953)	45.8209 (63.8214, 28.5420)
Western Europe	40,010.7218 (58,094.4089, 23,898.1557)	47,592.8502 (62,750.6267, 32,868.2534)	3.8687 (5.1345, 2.7236)	740,660.9565 (1,067,754.8335, 437,784.9898)	653,611.0378 (867,675.1637, 467,948.2500)	62.7878 (83.7768, 43.8507)
Southern Latin America	6,145.7245 (7,934.1232, 4,376.6786)	6,411.3309 (8,261.6474, 4,839.8133)	7.0174 (9.0411, 5.3117)	130,542.1713 (168,830.2952, 91,428.3049)	108,969.5531 (138,286.6303, 83,533.7402)	123.9193 (157.5016, 95.5633)
High-income North America	24,275.7403 (35,985.4615, 14,078.2497)	40,686.9473 (53,771.9987, 28,783.7982)	6.0751 (7.9835, 4.3560)	510,900.2247 (737,356.3138, 311,895.7812)	840,918.0242 (1,096,287.4296, 611,683.9732)	143.7869 (185.7280, 107.1345)
Caribbean	5,626.0086 (7,555.5688, 3,496.2893)	8,889.7830 (12,019.3597, 5,845.0592)	16.3363 (22.1255, 10.7008)	139,949.0413 (189,128.3689, 82,166.9739)	209,926.5241 (288,365.0531, 133,690.7729)	393.1281 (541.1831, 249.7927)
Andean Latin America	3,240.8778 (4,159.4402, 2,240.3891)	4,897.2932 (6,409.0702, 3,232.4917)	8.6617 (11.2853, 5.7487)	77,223.6463 (104,317.5368, 47,340.0603)	102,441.1602 (136,258.9218, 64,930.1498)	173.3587 (230.7839, 110.5029)
Central Latin America	10,694.8136 (13,968.1108, 7,329.8370)	19,170.2082 (26,151.6095, 12,566.9896)	8.0128 (10.9155, 5.2710)	254,181.8670 (335,774.7750, 167,427.3528)	411,464.7444 (561,934.1326, 270,669.3964)	164.4439 (224.1294, 108.5417)
Tropical Latin America	17,749.2751 (23,628.9915, 11,436.7219)	21,553.2163 (27,532.6075, 15,615.7258)	8.6384 (11.0134, 6.2791)	484,917.6638 (650,123.6603, 304,418.7862)	493,574.4457 (636,931.1977, 349,807.1492)	191.7606 (246.9934, 136.3690)
North Africa and Middle East	32,716.6910 (44,820.9241, 21,513.2202)	43,401.0041 (59,670.1577, 28,195.2821)	11.4894 (16.0275, 7.5847)	822,090.7342 (1,115,420.9870, 524,643.6730)	1,069,031.1346 (1,470,182.9842, 684,456.3046)	230.7791 (316.4253, 149.8559)
South Asia	102,136.5460 (143,164.1205, 60,671.5737)	198,595.2765 (267,168.5967, 125,107.4097)	15.2132 (20.5602, 9.9799)	2,976,316.4518 (4,165,709.1426, 1,653,027.9729)	5,134,319.9593 (6,971,118.4404, 3,059,858.0595)	341.4262 (461.0232, 209.1031)
Central Sub-Saharan Africa	10,314.7008 (14,878.3740, 6,039.2948)	20,693.4898 (29,323.6228, 11,749.0795)	48.7450 (68.0046, 29.1447)	295,913.5035 (430,809.4181, 164,691.4857)	570,451.1687 (818,666.7923, 302,389.4707)	1,030.0520 (1,446.9875, 591.3956)
Eastern Sub-Saharan Africa	32,392.7775 (42,983.0886, 20,110.2755)	44,022.6388 (59,712.6431, 27,356.6728)	32.1611 (43.1210, 21.1621)	907,581.9057 (1,211,391.6740, 543,074.3525)	1,202,352.0379 (1,637,591.3888, 706,153.8355)	683.6707 (924.5433, 423.6503)
Southern Sub-Saharan Africa	5,594.6111 (7,289.9690, 3,935.2627)	12,290.4431 (15,787.0614, 8,831.2815)	25.0647 (31.9441, 18.2655)	152,342.1571 (197,208.2773, 104,854.2514)	317,982.7543 (417,343.5575, 223,669.6474)	540.5510 (698.6712, 389.2716)
Western Sub-Saharan Africa	19,946.0997 (26,811.7201, 13,259.7773)	30,511.1732 (40,898.5461, 19,355.6476)	17.9160 (23.8491, 11.6906)	536,944.0576 (717,383.8712, 349,760.4946)	861,102.2773 (1,146,896.3731, 539,932.4245)	404.5444 (542.3140, 257.6374)
SDI						
High-middle SDI	91,217.8533 (124,235.7100, 60,656.8550)	78,002.5099 (112,621.8203, 50,402.0320)	4.1499 (5.9578, 2.6882)	2,076,618.0797 (2,827,216.4140, 1,364,820.2120)	1,392,169.0360 (2,012,151.1224, 888,266.2383)	73.2994 (105.4760, 46.9901)
High SDI	79,300.2218 (115,630.8686, 47,223.9242)	94,112.6216 (124,518.3016, 66,924.5396)	3.9992 (5.2800, 2.8278)	1,602,732.7401 (2,295,845.5701, 971,012.6577)	1,695,160.5070 (2,247,609.5393, 1,197,747.4624)	86.4299 (113.9092, 61.8907)
Low-middle SDI	127,290.7702 (174,490.9703, 76,219.8392)	201,905.9702 (269,254.3899, 132,037.9917)	15.9246 (21.2203, 10.6337)	3,593,289.9959 (4,934,850.7904, 2,035,669.5578)	5,171,329.3291 (6,920,685.0997, 3,254,156.5433)	353.4037 (470.8286, 226.2969)
Low SDI	73,340.0962 (98,675.2800, 44,947.7784)	118,766.6031 (158,541.1949, 74,849.1022)	27.7090 (36.4959, 18.3670)	2,065,802.6710 (2,789,592.2289, 1,192,509.5745)	3,269,665.9701 (4,407,207.7331, 1,970,522.0562)	613.8421 (820.9191, 384.8563)
Middle SDI	185,946.7316 (238,587.5488, 128,381.4672)	188,788.1493 (254,728.3050, 127,494.6844)	7.9384 (10.7879, 5.4157)	4,759,869.8711 (6,194,114.2586, 3,186,242.5360)	4,454,519.4584 (5,927,545.8637, 2,952,886.7597)	169.1608 (225.9855, 112.8965)

**Figure 1:**
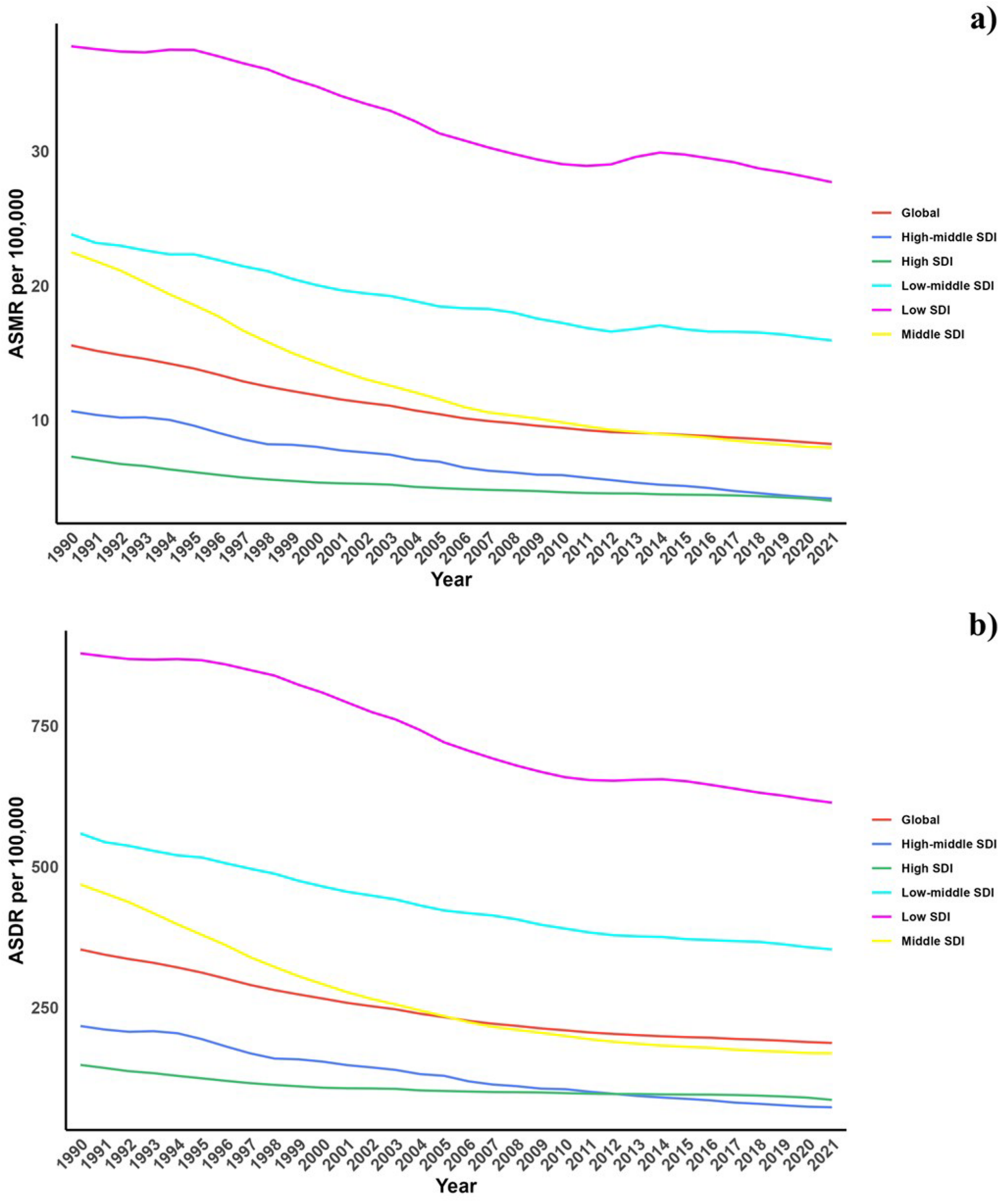
Temporal trends of ASMR and ASDR of CVD attributable to diet low in vegetables from 1990 to 2021 in different SDI regions.

Concerning the Socio-Demographic Index (SDI), significant declines in both ASMR and ASDR associated with diets low in vegetables were observed across all regions (Table 1, Supplementary Table S1, Figure 1).

Regionally, South Asia and Southeast Asia reported the highest burdens of CVD due to insufficient vegetable consumption in terms of deaths and DALYs. In contrast, Central, Eastern, and Southern Sub-Saharan Africa exhibited the highest ASMR and ASDR (Table 1).

**Figure 2:**
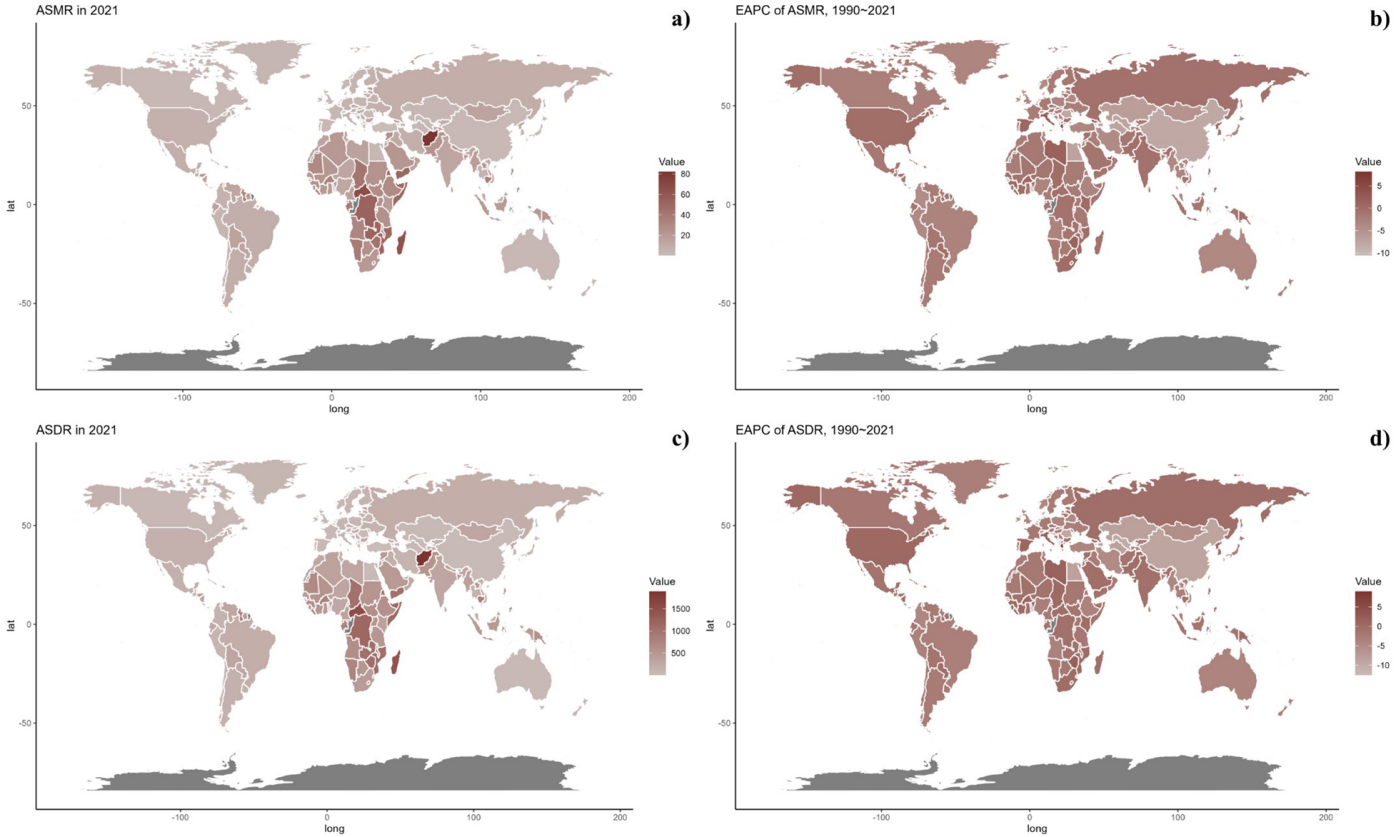
**(a)** Global distribution of ASMR of CVD attributable to Diet Low in Vegetables in 2021 in 204 countries and territories. **(b)** EAPC of ASMR of CVD attributable to Diet Low in Vegetables from 1990 to 2021 in 204 countries and territories. **(c)** Global distribution of ASDR of CVD attributable to Diet Low in Vegetables for both sexes in 2021 in 204 countries and territories. **(d)** EAPC of ASDR of CVD attributable to Diet Low in Vegetables from 1990 to 2021 in 204 countries and territories.

At the national level, the 2021 data highlighted substantial variations in ASMR and ASDR from CVDs related to low vegetable consumption, with the most elevated rates recorded in several African and South Asian countries. Over the period from 1990 to 2021, there has been a general uptick in both ASMR and ASDR within these regions (Table 1, Supplementary Table S1, Figure 2).

### 3.2 Age and gender pattern

**Figure 3:**
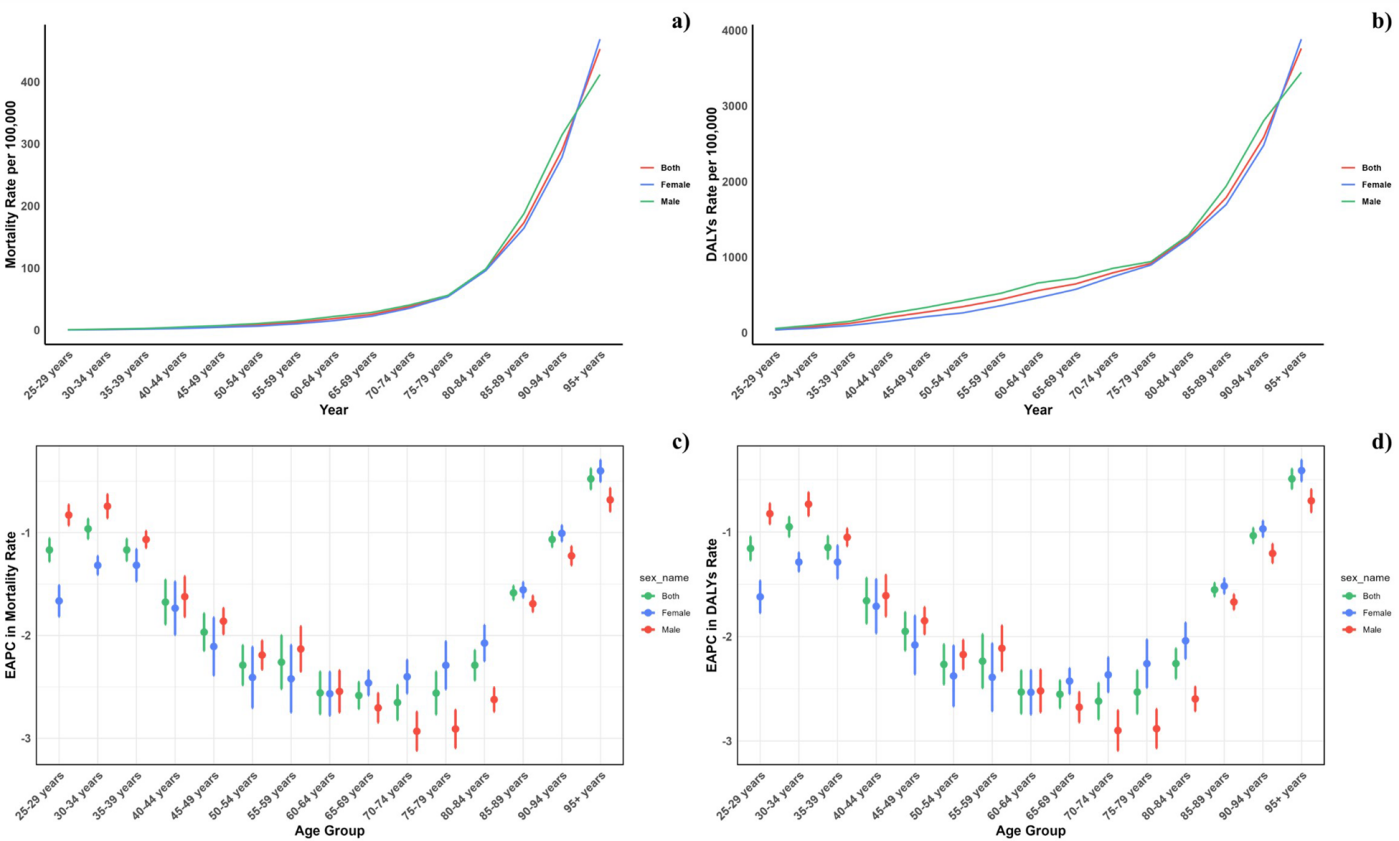
Age-specific rates of global deaths **(a)** and DALYs **(b)** of CVD attributable to diet low in vegetables, by sex, in 2021 and the corresponding EAPC of global deaths **(c)** and DALYs **(d)** from 1990 to 2021.

Figure 3 displays the global age-specific mortality and DALY rates for cardiovascular diseases (CVDs) in 2021, alongside historical trends from 1990. The graph illustrates a J-shaped curve, showing an increase in mortality and DALY rates for those under 75 years, with a pronounced rise for ages 75–95 and older. Throughout all age categories, mortality rates associated with low vegetable intake showed negligible gender differences, with DALY rates exhibiting a similar trend. From 1990 to 2021, the decrease in both mortality and DALY rates was more pronounced among females than males under 60 years.

**Figure 4:**
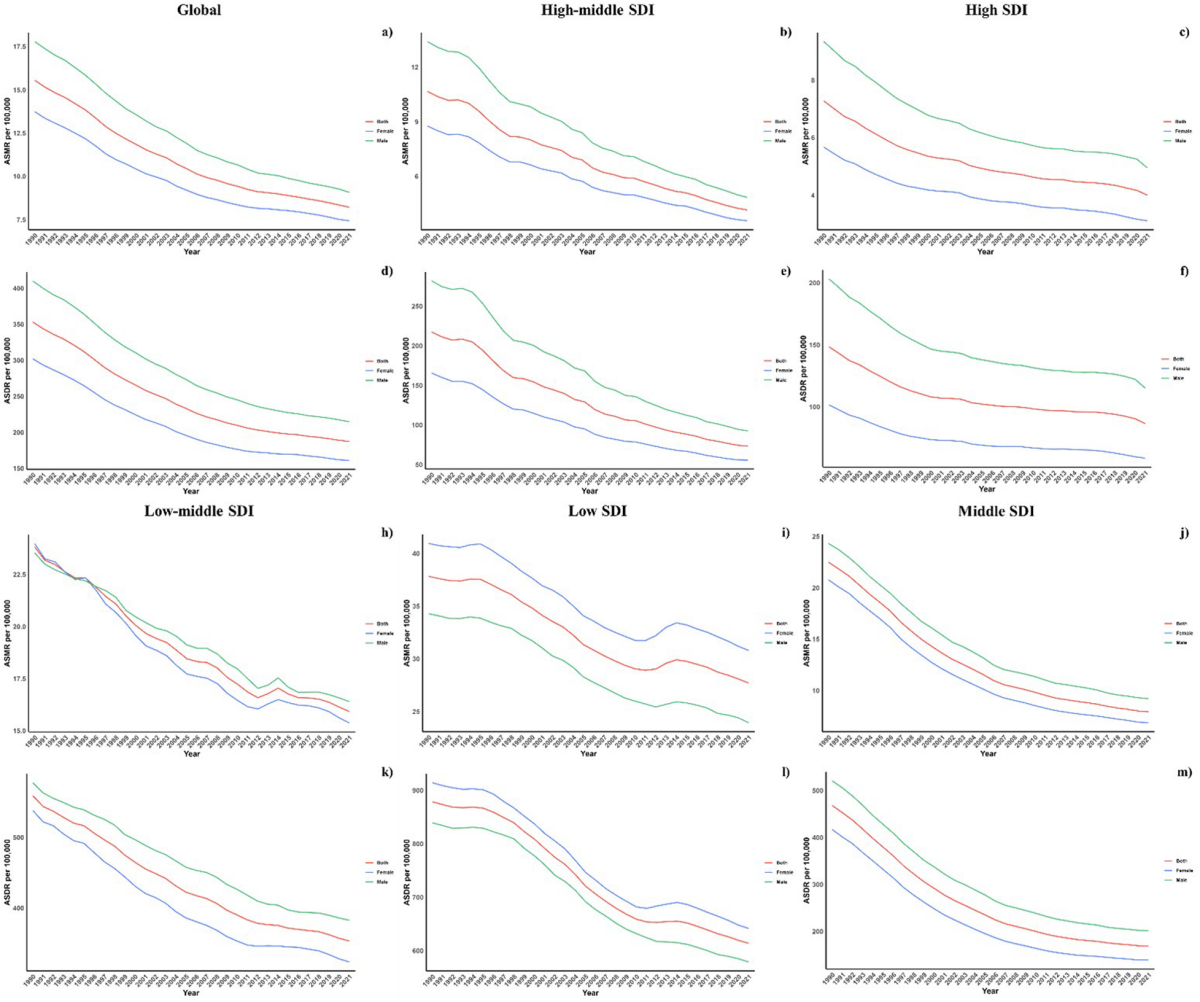
Sex disparity in CVD attributable to diet low in vegetables across SDI regions.

Across all Socio-Demographic Index (SDI) categories, mortality and DALY rates were generally higher in males, except in the low-middle SDI regions, highlighting a persistent gender disparity across various regions. However, this gap has lessened in high and high-middle SDI regions (Figure 4).

### 3.3 Association with the socio-demographic Index

**Figure 5:**
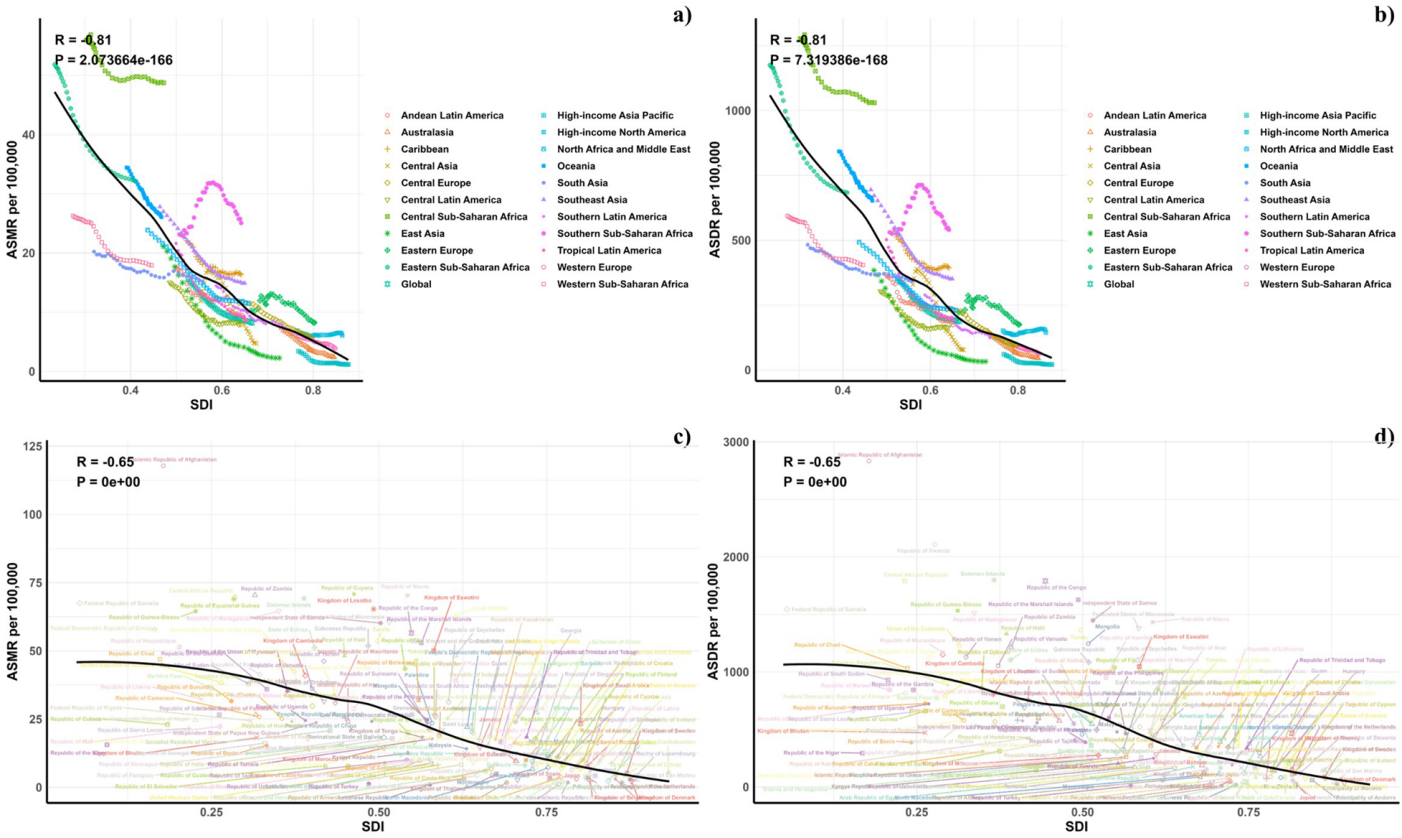
**(a)** Correlations between ASMR of CVD attributable to Diet Low in Vegetables and SDI at the regional level. **(b)** Correlations between ASDR of CVD attributable to Diet Low in Vegetables and SDI at the regional level. **(c)** Correlations between ASMR of CVD attributable to Diet Low in Vegetables and SDI at the national level. **(d)** Correlations between ASDR of CVD attributable to Diet Low in Vegetables and SDI at the national level.

Figure 5 presents a comparative analysis of the actual vs. projected age-standardized DALY (ASDR) and mortality rates (ASMR) for CVD due to low vegetable intake, related to Socio-Demographic Index (SDI) values at regional and national levels from 1990 to 2021. A marked negative correlation (R = −0.81, *P* < 0.0001) was observed between ASDR and rising SDI, suggesting a heavier disease burden in lower SDI areas. Regions such as Southeast Asia, Central Sub-Saharan Africa, and Oceania reported ASDRs that were higher than expected during this timeframe. The patterns of observed vs. forecasted ASMR based on SDI at the regional level corresponded with the ASDR findings. Additionally, Figure 5 illustrates the observed and projected ASDR and ASMR for 2021 at the national level, demonstrating a similar inverse relationship with SDI across both regional and national contexts.

### 3.4 Forecasts for the mortality, DALYs rate, ASMR and ASDR of CVD attributable to diet low in vegetables in global (2022–2050)

**Figure 6:**
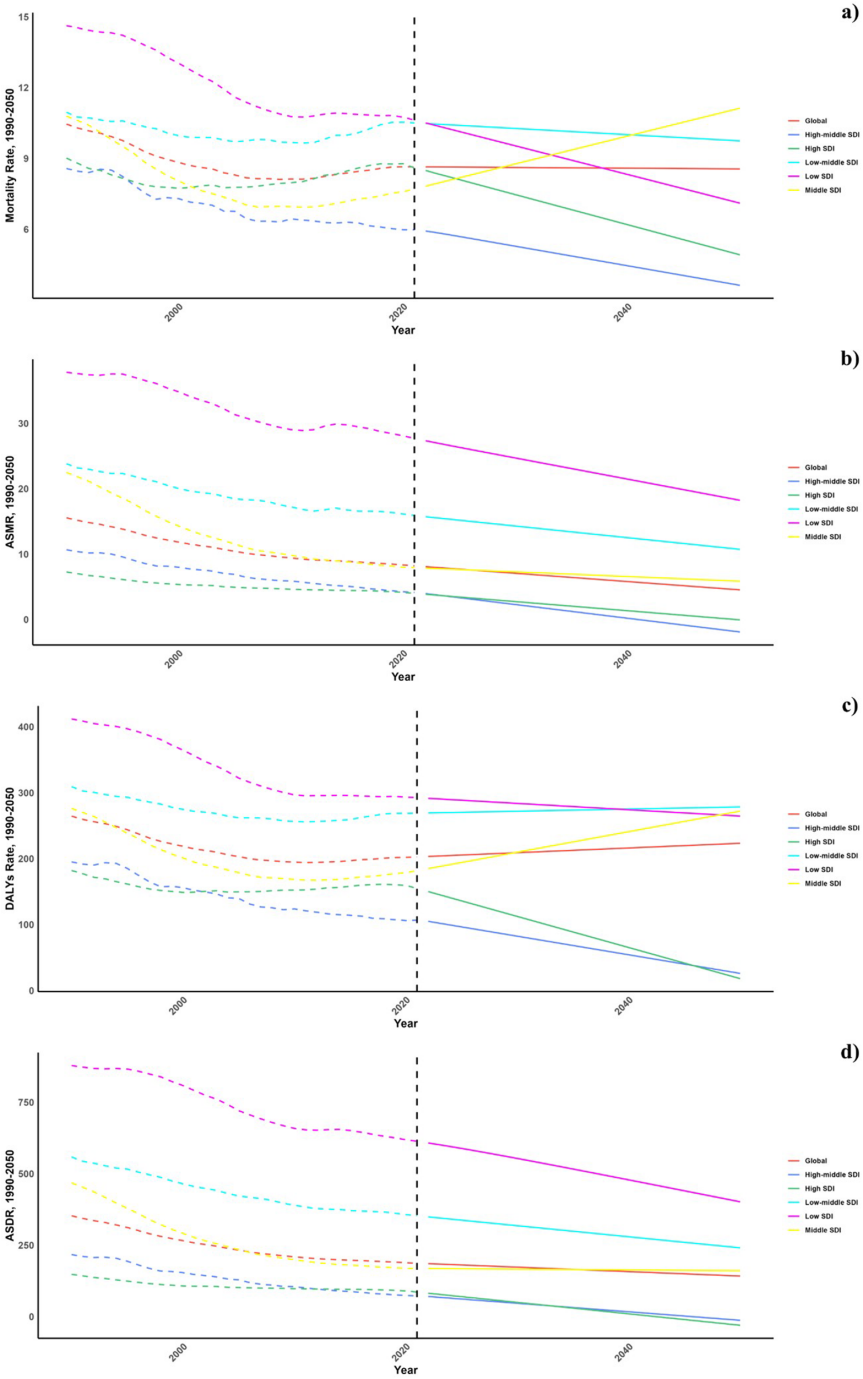
Estimated trends of mortality rate **(a)**, DALYs rate **(b)**, ASMR **(c)** and ASDR **(d)**, 1990–2050 at the regional level.

**Figure 7:**
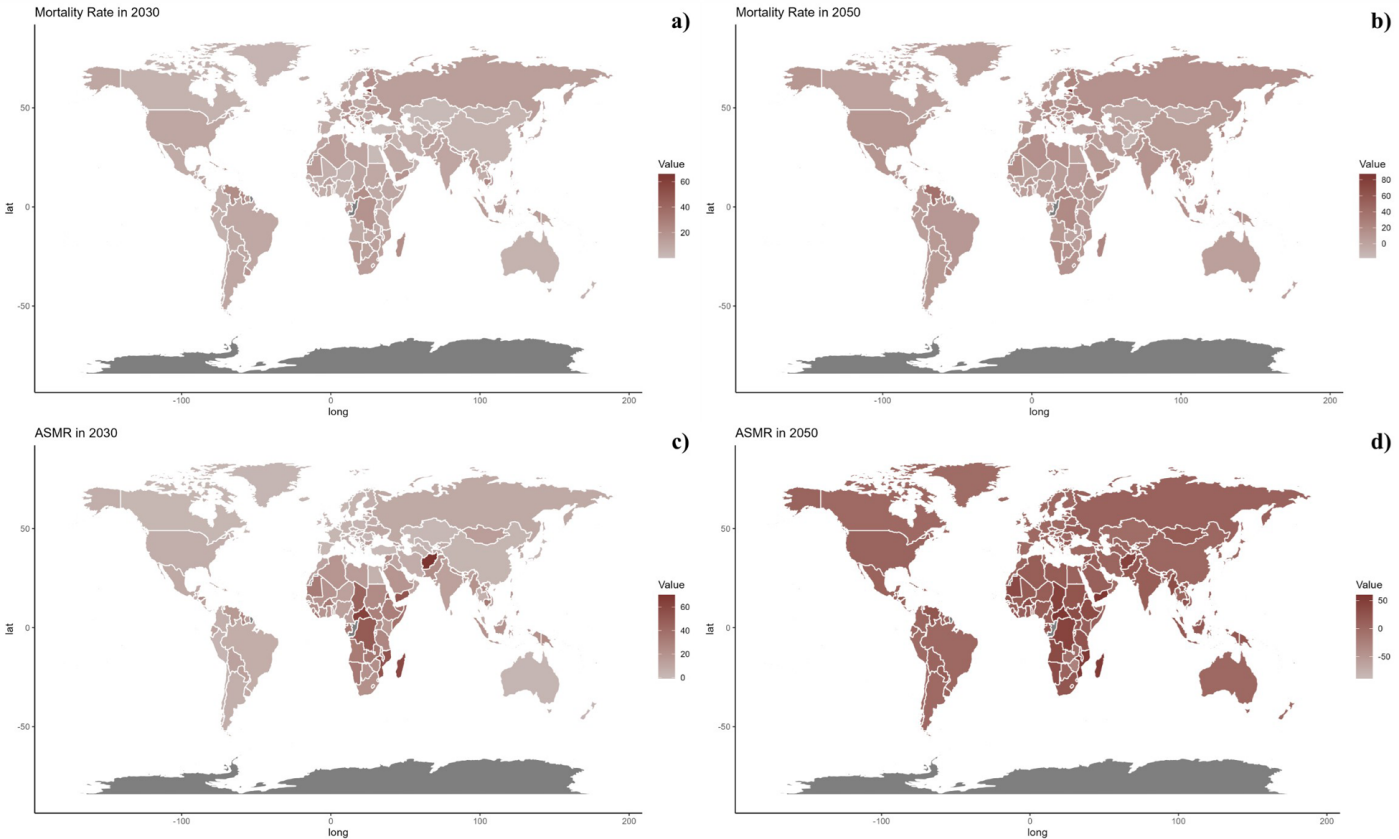
Estimated trends of mortality rate **(a, b)** and ASMR **(c, d)** in 2030 **(a, c)** and 2050 **(b, d)** at the national level.

**Figure 8:**
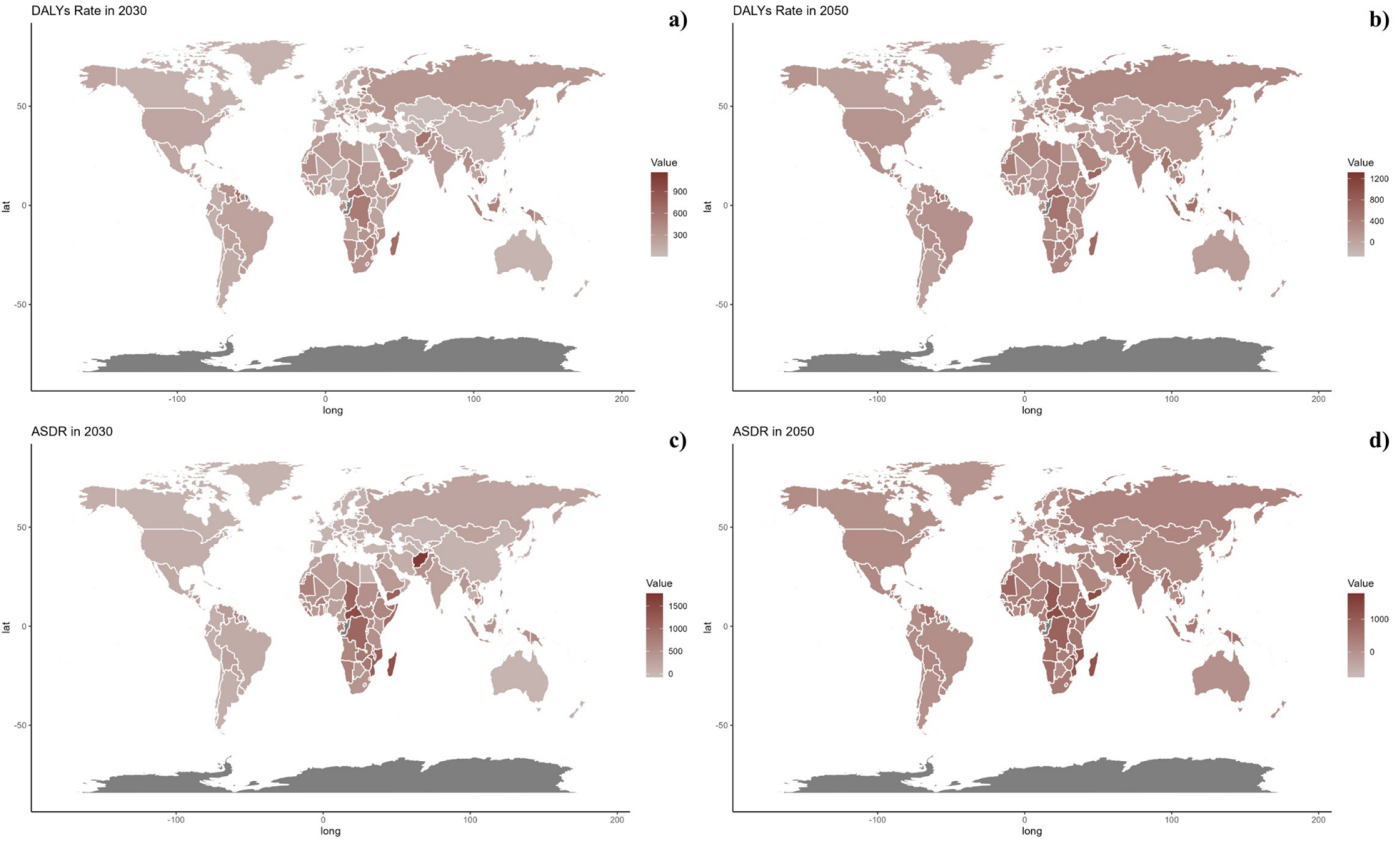
Estimated trends of DALYs rate **(a, b)** and ASDR **(c, d)** in 2030 **(a, c)** and 2050 **(b, d)** at the national level.

Future projections for mortality and DALY rates, including age-standardized mortality rate (ASMR) and age-standardized DALY rate (ASDR), associated with CVDs due to vegetable consumption are depicted in Figures 6–8. Most regions, categorized by the Socio-Demographic Index (SDI), are anticipated to observe a decline in the CVD burden, with the exception of low-middle SDI regions where increases in both mortality and DALY rates are expected.

At the national level, projections indicate that trends are likely to remain consistent through 2030 and 2050. Nonetheless, African countries are projected to encounter significantly higher burdens of CVD related to inadequate vegetable intake during these years.

## 4 Discussion

This research assesses the global effects of cardiovascular diseases (CVD) related to insufficient vegetable consumption from 1990 to 2021, revealing a significant decline in cases throughout this period. Individuals over 75 were most impacted. Future projections suggest increasing CVD burdens in lower-middle Socio-Demographic Index (SDI) areas, with consistently high burdens expected in African regions. This study offers a comprehensive examination of the consequences and future projections of vegetable deficits on CVD.

Many studies have examined the link between fruit and vegetable consumption and CVD risk, typically identifying positive effects. A recent comprehensive meta-analysis of cohort studies involving 4,031,896 participants across 69 countries found that those with the highest intake levels had a 7%, 9%, and 6% reduced risk of CVD for total fruits and vegetables, fruits, and vegetables respectively, compared to those with the lowest intake levels (7). These findings align with substantial evidence suggesting that higher consumption of fruits and vegetables is associated with a lower risk of CVD. However, research in different geographical settings, particularly in Asian populations, has shown varied results. In line with our observations, the China-PAR study indicated that those in the highest quintile of total fruit and vegetable consumption had a 15% decreased CVD risk [0.85 (0.77, 0.95)] (16). This was consistent with findings that greater fruit consumption was associated with an 18% reduction in CVD risk [0.82 (0.74, 0.91)], although no significant correlation was found for vegetables alone. These results highlight the possibility that the effect of fruits and vegetables on CVD may be influenced by the type of produce consumed, as well as other factors such as lifestyle habits and cultural dietary patterns.

Conversely, Takachi et al. reported no link between total fruit and vegetable consumption and CVD risk in Japanese cohorts but did identify an inverse relationship between fruit consumption and CVD risk [0.81 (0.67, 0.97)] (14). Such discrepancies may be attributed to the differences in dietary patterns, food culture, and genetic factors across populations. For instance, dietary habits in Japan may involve high consumption of other protective foods, such as fish and soy-based products, that could confound the relationship between fruit and vegetable intake and CVD risk. The causes for these differing outcomes remain unclear, and further research is needed to better understand how fruit and vegetable consumption interacts with other dietary and lifestyle factors to influence cardiovascular health across diverse populations.

Proposed mechanisms suggest that fruit and vegetable consumption may decrease CVD risk due to their rich content of beneficial nutrients, such as dietary fiber, plant proteins, vitamin C, minerals, polyphenols, phytoestrogens, and carotenoids, all of which are known to aid in CVD prevention (34). Dietary fiber, for example, can regulate gut microbiota, enhancing the production of short-chain fatty acids (SCFAs) that contribute to cardiovascular health by lowering systemic inflammation and inhibiting platelet aggregation (35). Additionally, the antioxidants found in fruits and vegetables, particularly vitamin C, are associated with lower incidences of CVD, including hypertension, coronary heart disease (CHD), heart failure, and stroke (36). Vitamin C's antioxidant properties are thought to protect blood vessels by reducing oxidative stress, which is a key factor in the development of atherosclerosis and other cardiovascular conditions. Plant proteins, commonly found in legumes, nuts, and seeds, are also linked to reduced CVD risk by lowering cholesterol levels and reducing inflammation (37). Moreover, polyphenols present in a variety of fruits and vegetables, such as those found in soybeans and grapes, including phenolic acids and flavonoids, are believed to support cardiovascular health through their anti-inflammatory, antioxidant, and gut microbiota-regulating effects (38). These bioactive compounds may contribute to the prevention of CVD by improving endothelial function, reducing arterial stiffness, and modulating the balance of pro- and anti-inflammatory molecules in the body.

Despite these promising mechanisms, the precise pathways through which fruits and vegetables reduce CVD risk are complex and multifactorial. Additional studies are needed to further explore these mechanisms and how they may vary based on the type of fruits and vegetables consumed, as well as individual genetic factors and overall dietary patterns.

The policy implications of our study emphasize the need for healthcare system strengthening, particularly in low- and middle-income countries (LMICs), where the CVD burden is highest. Policy actions should focus on improving access to CVD prevention, early diagnosis, and treatment, with an emphasis on primary care in underserved regions. Lifestyle interventions, including healthier food policies and tobacco control, should be prioritized globally. Addressing social determinants of health and reducing health inequities are essential for long-term prevention. Additionally, adapting healthcare systems to the needs of aging populations and fostering cross-sector collaboration will be crucial for mitigating the future CVD burden.

While vegetable consumption plays a protective role in CVD due to its high content of fiber, vitamins, and antioxidants, high intake of salt, saturated fats, and refined sugars remains a significant global risk factor. Reducing these harmful dietary factors, while increasing vegetable intake, is essential for mitigating the CVD burden. A balanced, plant-based diet can provide synergistic benefits, making it a key strategy in CVD prevention.

Implementing dietary interventions to reduce cardiovascular disease (CVD) burden faces several challenges, including cultural and behavioral factors, economic constraints, limited access to healthy foods, and gaps in health literacy. In low- and middle-income countries (LMICs), the high cost and limited availability of vegetables pose significant barriers, while in high-income countries, economic inequality and food deserts contribute to disparities in access to nutritious foods. Public health campaigns, education on the benefits of vegetables, and policies to make healthy foods more affordable are critical. Additionally, integrating dietary interventions into healthcare systems and addressing policy barriers is key for successful implementation.

Nonetheless, this study encounters several limitations. The lack of data from specific countries could introduce bias into our findings. The detail on total and daily vegetable intake was also insufficient, limiting the analysis. The variety of fruits assessed was too narrow to comprehensively evaluate CVD risks. Moreover, there were gaps in the data concerning mortality and DALY trends from 1990 to 2021. Critically, there was a lack of data on individuals under 25 years old, a demographic notably characterized by low fruit consumption.

## 5 Conclusion

This study offers an in-depth evaluation of the worldwide effects of cardiovascular diseases (CVD) associated with insufficient vegetable consumption from 1990 to 2021, highlighting a notable reduction in cases during this period. Individuals aged 75 and older were predominantly impacted. Future forecasts suggest that mortality and DALY rates will rise in regions with low-middle SDI. Nationally, it is anticipated that African countries will continue to experience significant CVD challenges linked to low vegetable intake up to 2030 and 2050.

These findings are crucial for the development of strategies aimed at preventing CVD and emphasize the importance of managing dietary factors.

## Data Availability

The raw data supporting the conclusions of this article will be made available by the authors, without undue reservation.
