# Supplementary material for: Global burden of cardiovascular diseases attributable to diet low in vegetables from 1990 to 2021 and forecasting the future trends: a population-based study
Source: Front Cardiovasc Med. 2025 Jan 15;11:1491869. doi: 10.3389/fcvm.2024.1491869 (PMC11774848; doi:10.3389/fcvm.2024.1491869)
Supplement: Supplementary file 1 [file Table1.docx]

Table S1 Global and regional deaths and DALYs of CVD Attributable to Diet Low in Vegetables in 1990 and 2021 in 204 nations

| Location | Deaths Number in 1990 | Deaths Number in 2021 | ASMR in 2021 | DALY Number in 1990 | DALY Number in 2021 | ASDR in 2021 |
| --- | --- | --- | --- | --- | --- | --- |
| People's Republic of China | 124165.1867 (161716.8919, 88271.2249) | 32251.2314 (58923.6341, 13212.2763) | 2.0321 (3.7422, 0.8160) | 2870500.7412 (3771634.2626, 2073499.2359) | 481279.4420 (894442.6488, 204982.9187) | 27.6628 (50.7346, 11.8749) |
| Democratic People's Republic of Korea | 1334.3546 (2041.1834, 806.4891) | 4132.4393 (6043.9921, 2647.2377) | 14.4798 (21.0646, 9.3019) | 29431.0821 (44265.5268, 17806.5785) | 89159.7054 (129143.9920, 56392.8328) | 284.0854 (407.5694, 181.1756) |
| Taiwan (Province of China) | 1292.8513 (1614.6060, 987.0489) | 1832.1031 (2502.1658, 1227.1250) | 4.0144 (5.4593, 2.6523) | 29517.1863 (36929.6922, 22559.6724) | 30088.5303 (42059.9097, 19742.4483) | 70.9867 (99.1429, 46.3724) |
| Kingdom of Cambodia | 1919.8098 (2807.7871, 1062.7238) | 3856.7144 (5579.8938, 2197.9988) | 36.0527 (51.9169, 21.2537) | 55017.9743 (79598.3894, 28360.1711) | 102524.0312 (149401.5483, 55904.7392) | 810.4837 (1173.3001, 458.4001) |
| Republic of Indonesia | 26739.3329 (39072.4337, 12546.8478) | 40113.0999 (55970.0042, 23136.9644) | 19.5826 (27.4728, 11.4233) | 853517.2197 (1252664.1819, 361807.4954) | 1148373.8021 (1619237.7267, 656022.2386) | 454.9142 (630.7768, 260.9230) |
| Lao People's Democratic Republic | 1490.6139 (2212.5852, 714.7797) | 925.4469 (1291.4590, 606.6298) | 24.1589 (33.6265, 15.9136) | 45100.3222 (67523.6105, 20832.8496) | 24701.6485 (34541.0404, 15901.6004) | 516.2781 (717.0247, 335.8435) |
| Malaysia | 1806.4126 (2723.2185, 776.1310) | 2287.3366 (3303.3488, 1282.5495) | 8.7521 (12.7305, 4.8487) | 52344.6063 (78721.4487, 20871.2501) | 61796.7265 (88044.1866, 35305.9662) | 210.5297 (301.0323, 119.6462) |
| Republic of Maldives | 15.4784 (22.0191, 8.7384) | 18.8507 (25.9960, 12.0639) | 6.2969 (8.6940, 4.1320) | 478.5901 (683.1982, 259.1161) | 485.3678 (658.1444, 305.7975) | 125.8156 (171.5867, 81.2323) |
| Republic of the Union of Myanmar | 9981.5972 (14960.0229, 5230.0064) | 7309.1743 (10512.5678, 4475.3547) | 17.2109 (24.7212, 10.4381) | 298065.6566 (449960.5758, 143091.5375) | 183050.7586 (261203.1475, 113509.0821) | 375.2768 (536.3271, 233.4959) |
| Republic of the Philippines | 7267.1851 (9599.2846, 5040.9747) | 17553.2951 (22638.1636, 12855.0383) | 24.2330 (31.2187, 17.8971) | 206033.6149 (271941.1762, 138660.1376) | 471517.4237 (620989.8922, 339578.6475) | 551.0572 (713.3295, 401.1965) |
| Democratic Socialist Republic of Sri Lanka | 2784.2391 (3775.7788, 1727.5257) | 2824.2492 (4338.4832, 1654.6017) | 11.5517 (17.8271, 6.5988) | 74177.5274 (99884.3703, 46222.4328) | 65335.4027 (97559.9368, 37901.6062) | 249.7420 (374.7349, 146.4379) |
| Kingdom of Thailand | 3784.7165 (5876.6707, 1625.7608) | 5250.6575 (7759.9914, 2813.9932) | 4.9232 (7.2833, 2.6387) | 110743.9975 (168204.2380, 44110.0710) | 130473.0810 (190501.7719, 70171.9347) | 127.6089 (187.3661, 68.1725) |
| Democratic Republic of Timor-Leste | 105.0520 (156.9199, 51.1668) | 268.5741 (407.9962, 135.9265) | 35.4498 (53.0557, 18.3054) | 3289.8205 (4959.9046, 1475.5914) | 6969.3589 (10665.2388, 3396.3281) | 806.3291 (1218.4914, 401.5395) |
| Socialist Republic of Viet Nam | 9192.5606 (13199.9593, 5007.1187) | 7678.4774 (11209.1508, 4969.2383) | 9.1943 (13.3640, 5.9356) | 226459.0927 (327233.1511, 115102.8691) | 171214.0711 (250071.5804, 108070.4122) | 178.9527 (259.9708, 115.1283) |
| Republic of Fiji | 148.3382 (208.5815, 80.3613) | 164.2633 (239.7305, 97.2480) | 25.3505 (36.2311, 15.3395) | 4889.4402 (6953.0447, 2523.2081) | 4786.0015 (7093.3417, 2768.1859) | 600.7141 (873.1812, 354.6991) |
| Republic of Kiribati | 10.8743 (16.0660, 4.9994) | 18.9148 (29.3729, 8.8416) | 27.4167 (41.5875, 13.7561) | 365.5475 (544.6211, 154.1815) | 647.8104 (1014.2413, 286.0250) | 753.5122 (1166.7689, 350.6980) |
| Republic of the Marshall Islands | 9.6352 (13.7585, 4.7848) | 16.5963 (25.1549, 8.1867) | 51.4317 (75.8052, 27.6882) | 300.2749 (430.3928, 141.6380) | 564.7672 (860.4465, 268.8409) | 1348.5028 (2023.3149, 673.6671) |
| Federated States of Micronesia | 31.1548 (44.8395, 15.9022) | 35.3478 (53.2234, 18.1697) | 52.6124 (78.4922, 29.6333) | 947.9767 (1385.7781, 447.1104) | 1127.5130 (1719.9632, 572.2011) | 1367.3107 (2043.4837, 702.4873) |
| Independent State of Papua New Guinea | 400.6808 (589.0532, 211.0862) | 1064.0993 (1560.7586, 556.0566) | 23.2908 (34.2939, 12.6158) | 12512.2917 (18498.0883, 6488.4211) | 33574.4622 (49363.7172, 17618.8552) | 570.2344 (830.3017, 302.8332) |
| Independent State of Samoa | 48.2287 (70.5868, 23.0987) | 66.3331 (97.7616, 33.2995) | 49.3669 (72.0209, 25.3059) | 1391.6945 (2079.9889, 614.3868) | 1912.4326 (2875.4357, 931.4482) | 1250.2879 (1863.9328, 619.7717) |
| Solomon Islands | 93.9940 (145.6662, 36.3608) | 224.0261 (351.6940, 91.6716) | 67.4642 (104.0267, 29.3354) | 3031.3051 (4739.7706, 1118.0374) | 7366.3261 (11553.8464, 2960.1113) | 1781.0639 (2781.6398, 737.4040) |
| Kingdom of Tonga | 10.4215 (15.6608, 4.9005) | 12.3703 (18.6188, 6.0123) | 15.7623 (23.7772, 7.7477) | 303.5105 (454.9669, 140.0950) | 328.7985 (495.5491, 150.9089) | 399.0495 (601.3442, 184.8044) |
| Republic of Vanuatu | 22.4240 (35.0360, 9.4790) | 57.5548 (86.3241, 27.1487) | 34.5365 (51.4544, 16.5918) | 760.5177 (1198.7937, 308.8472) | 1939.2746 (2897.1661, 893.3472) | 937.5735 (1395.3716, 441.5047) |
| Republic of Armenia | 149.2524 (248.0535, 72.4507) | 26.8468 (78.7502, 2.1278) | 0.6308 (1.8180, 0.0577) | 2820.2470 (4906.8371, 1306.9045) | 295.2962 (827.6462, 23.6375) | 6.8709 (19.0235, 0.6109) |
| Republic of Azerbaijan | 678.3507 (1006.7056, 410.0176) | 241.2702 (475.2084, 83.5470) | 3.3915 (6.7435, 1.2076) | 15880.3714 (23773.7965, 9554.8140) | 4409.7455 (9231.8242, 1444.0722) | 52.9191 (107.1373, 18.3735) |
| Georgia | 656.3929 (1002.0230, 362.2725) | 1017.5634 (1384.1457, 704.8758) | 15.6462 (21.0926, 10.8513) | 14488.0874 (22041.0669, 8066.9744) | 17786.3863 (23847.0375, 12333.7837) | 299.1556 (401.1288, 210.4827) |
| Republic of Kazakhstan | 1378.2088 (2050.2265, 770.3566) | 388.0238 (750.2887, 155.3763) | 3.2024 (5.9565, 1.3489) | 36032.6725 (52352.3466, 20995.4151) | 7204.6930 (14273.9680, 2736.1537) | 48.9280 (94.6926, 19.6786) |
| Kyrgyz Republic | 260.4190 (392.2491, 152.5383) | 291.2060 (471.8922, 149.8471) | 7.6507 (12.1385, 4.0211) | 6282.6450 (9422.2763, 3752.0130) | 6316.8889 (10471.7594, 3217.8487) | 140.7331 (227.0588, 72.3789) |
| Mongolia | 474.3273 (758.6056, 165.4368) | 257.4093 (381.7065, 127.9448) | 14.3181 (21.3819, 7.1047) | 12702.5413 (20265.7788, 4170.1922) | 6934.4954 (10362.7824, 3415.8047) | 295.0496 (434.7769, 146.5931) |
| Republic of Tajikistan | 402.5149 (612.3991, 231.7294) | 384.5788 (629.3795, 205.2926) | 10.0721 (16.1247, 5.5809) | 8268.0554 (12051.1476, 4913.5561) | 8256.1427 (13896.4130, 4207.2054) | 166.1928 (269.4595, 87.3422) |
| Turkmenistan | 219.8879 (331.4848, 123.3325) | 81.9524 (167.9772, 24.6169) | 2.7087 (5.5803, 0.8783) | 5441.1555 (8206.1470, 3101.6342) | 1543.2831 (3407.4600, 429.1800) | 44.0140 (91.8397, 13.1710) |
| Republic of Uzbekistan | 795.4242 (1302.7254, 397.1969) | 200.4388 (461.3271, 40.8460) | 1.3829 (3.1874, 0.3046) | 17895.2294 (29132.6809, 8863.8332) | 2547.4450 (6207.3245, 494.8289) | 15.5591 (36.1907, 3.1485) |
| Republic of Albania | 153.1100 (217.6510, 92.3067) | 62.9477 (124.8510, 19.8927) | 1.7032 (3.3572, 0.5550) | 3349.1135 (4749.1785, 2000.9349) | 820.3298 (1725.0192, 253.8548) | 21.2406 (43.2700, 6.8493) |
| Bosnia and Herzegovina | 374.9299 (539.5118, 226.0003) | 305.8431 (481.2768, 160.2110) | 4.8225 (7.5957, 2.5531) | 9172.1807 (13128.6813, 5554.4795) | 5114.5612 (8040.4476, 2698.5383) | 82.9915 (130.1653, 43.0039) |
| Republic of Bulgaria | 1355.0905 (2045.0579, 832.4887) | 2090.3888 (3366.6776, 1098.3232) | 15.4838 (24.3229, 8.4449) | 29706.6989 (44873.1114, 17897.6286) | 29166.2732 (47450.0157, 14832.5496) | 216.1493 (358.3401, 111.3053) |
| Republic of Croatia | 843.8067 (1177.2826, 542.5263) | 708.2768 (1021.5854, 434.8157) | 7.3407 (10.4672, 4.5320) | 15990.5394 (22487.8417, 10225.4602) | 11248.5661 (15948.9943, 6984.8673) | 126.7576 (178.4190, 79.8697) |
| Czech Republic | 1759.6619 (2643.8675, 890.1561) | 1744.8204 (2396.6535, 1114.8973) | 7.5669 (10.4124, 4.8030) | 38958.5428 (57544.2996, 20594.1831) | 29168.8334 (40515.6226, 18896.4773) | 136.0760 (187.5117, 87.7355) |
| Hungary | 2056.9747 (2903.1913, 1311.0749) | 1608.2099 (2344.3207, 1019.3131) | 7.5547 (11.0116, 4.7954) | 46462.8093 (65183.5830, 29593.1357) | 26537.3571 (39086.7756, 16586.1869) | 136.8375 (201.6640, 84.1571) |
| North Macedonia | 96.7939 (164.2775, 47.6112) | 99.1075 (196.1255, 34.7022) | 5.0758 (9.6840, 2.0226) | 1688.9572 (2965.9537, 769.1846) | 1542.9315 (3085.1057, 548.2616) | 65.6117 (126.5261, 25.8337) |
| Montenegro | 12.8625 (23.3682, 5.2821) | 15.1453 (30.3781, 5.2522) | 2.1271 (4.1625, 0.8093) | 244.6743 (464.9299, 94.8130) | 203.3890 (421.2673, 70.1282) | 26.0911 (51.2336, 9.5725) |
| Republic of Poland | 3931.4220 (5906.7537, 2252.1575) | 3145.3766 (4908.7813, 1796.5773) | 3.9894 (6.1604, 2.2809) | 88530.4689 (133240.8135, 50643.8330) | 48288.8333 (73772.7376, 27834.1506) | 65.7931 (100.5360, 38.3742) |
| Romania | 2091.4002 (3221.0409, 1192.5886) | 490.8628 (1177.3262, 83.2576) | 1.1432 (2.6875, 0.1992) | 40272.0243 (63925.3523, 22647.5749) | 5833.3986 (13965.2278, 1014.7291) | 14.1753 (33.6867, 2.6266) |
| Republic of Serbia | 1548.9026 (2238.6137, 1033.0192) | 1892.9765 (2735.2450, 1184.2622) | 11.0715 (15.9973, 6.9685) | 31146.3879 (44526.6520, 20359.5149) | 30486.2569 (44054.2882, 19219.3414) | 185.3228 (265.4166, 117.4060) |
| Slovak Republic | 828.1297 (1221.8823, 463.2596) | 755.6768 (1086.5554, 439.0902) | 8.0283 (11.5121, 4.6618) | 18552.5513 (26950.3094, 10644.0897) | 13565.3820 (19445.8378, 7952.8930) | 146.2714 (207.9929, 86.0648) |
| Republic of Slovenia | 240.5921 (335.6819, 163.0266) | 356.7841 (485.2310, 232.0326) | 6.2683 (8.4184, 4.1852) | 4901.3103 (6686.8616, 3310.8580) | 4778.2530 (6429.5604, 3273.6116) | 95.3267 (128.0418, 65.7804) |
| Republic of Belarus | 1488.0090 (2271.4247, 746.4044) | 910.6049 (1621.5279, 357.1211) | 5.5836 (9.9409, 2.2275) | 33487.8269 (49515.3767, 17784.9556) | 15712.9544 (28207.1320, 6212.1357) | 100.0614 (180.5656, 39.2419) |
| Republic of Estonia | 362.1276 (523.2305, 209.2006) | 739.7748 (1034.8465, 500.9099) | 21.7127 (29.9592, 14.9956) | 8103.6574 (11533.1396, 4878.7599) | 9888.1447 (13455.3769, 6889.9464) | 323.5025 (443.6631, 230.2654) |
| Republic of Latvia | 392.7654 (614.1065, 189.8957) | 410.4173 (593.6708, 264.1746) | 9.0566 (12.9811, 5.8179) | 8607.4321 (13126.9992, 4341.9939) | 6843.8129 (9757.9778, 4422.8456) | 173.0360 (244.8121, 112.4928) |
| Republic of Lithuania | 587.1153 (916.1422, 289.0361) | 497.5476 (748.7167, 280.4089) | 7.5510 (11.3511, 4.2744) | 12598.8104 (18891.3814, 6703.5042) | 8158.3628 (12178.9075, 4699.1289) | 142.4576 (212.3887, 83.5022) |
| Republic of Moldova | 369.0528 (585.1634, 179.9307) | 917.0806 (1250.1169, 628.9815) | 15.2397 (20.7481, 10.4121) | 8065.2813 (12562.7309, 4152.3359) | 17671.4114 (24147.1583, 12009.7202) | 299.6627 (410.3542, 201.6137) |
| Russian Federation | 16435.0314 (25252.5576, 8493.4510) | 20027.2889 (30238.9034, 11183.2875) | 8.4025 (12.6051, 4.7120) | 391561.9002 (590516.5467, 208556.3682) | 437724.9003 (647073.9000, 243595.0558) | 190.3084 (279.9913, 107.2003) |
| Ukraine | 6185.8137 (9719.5084, 3100.1269) | 5653.0914 (9981.4766, 2473.5183) | 7.1792 (12.6442, 3.1050) | 130720.5454 (203267.8667, 69414.5775) | 98135.2423 (172384.0332, 42403.0976) | 129.0906 (226.1413, 55.9154) |
| Brunei Darussalam | 14.4662 (19.4356, 9.6836) | 25.4188 (34.4417, 17.4997) | 10.5542 (13.8305, 7.4917) | 405.5350 (545.9490, 269.1583) | 728.9182 (983.4293, 481.2969) | 206.4655 (275.9383, 141.7391) |
| Japan | 4888.3165 (7067.9385, 3132.4352) | 5317.8506 (8395.5803, 2785.2632) | 0.9192 (1.4075, 0.5184) | 93907.5228 (134315.5869, 60360.0307) | 69405.6967 (107741.5324, 39325.9965) | 17.9788 (27.1768, 10.7791) |
| Republic of Korea | 680.7966 (1083.1820, 368.2504) | 2601.5747 (3897.9256, 1612.9066) | 3.0622 (4.5732, 1.8863) | 12850.2227 (21931.5379, 6130.0553) | 33637.6084 (52483.4613, 21098.4778) | 38.8780 (59.6625, 24.3055) |
| Republic of Singapore | 202.1761 (276.6033, 138.7643) | 262.7314 (356.2754, 174.8675) | 3.1514 (4.2682, 2.0814) | 5015.0124 (6749.4933, 3398.4941) | 5408.5980 (7410.1677, 3606.1294) | 63.6078 (86.9881, 42.7301) |
| Australia | 1382.7725 (2054.8220, 720.1460) | 1231.1335 (1753.3200, 754.2059) | 2.3613 (3.3421, 1.4505) | 28260.3061 (41177.3650, 15182.1345) | 20193.1901 (28088.4376, 12464.2065) | 44.7983 (62.0239, 27.8642) |
| New Zealand | 277.9158 (417.0996, 144.0389) | 246.0067 (368.7994, 140.7725) | 2.7072 (4.0437, 1.5480) | 5922.2530 (8800.7272, 3172.1461) | 4216.2426 (6098.6113, 2487.8115) | 51.1239 (73.4605, 30.2843) |
| Principality of Andorra | 2.2393 (3.6228, 1.2504) | 5.3479 (8.2999, 3.1637) | 2.8853 (4.4576, 1.7164) | 45.1929 (73.6411, 24.8605) | 77.5113 (118.2991, 46.6727) | 46.3389 (69.7858, 28.1628) |
| Republic of Austria | 1197.8787 (1683.8501, 724.2860) | 1242.9719 (1690.3390, 833.3804) | 5.2584 (7.1520, 3.5315) | 22211.0835 (31115.8284, 13447.0103) | 16743.3929 (23007.3967, 11439.8274) | 80.9956 (111.1439, 54.8908) |
| Kingdom of Belgium | 830.4927 (1255.5935, 445.3565) | 415.6676 (646.9374, 241.1891) | 1.3925 (2.1516, 0.7988) | 15952.1154 (24212.1609, 8540.5579) | 6146.9254 (9523.9317, 3357.0243) | 24.7847 (38.3382, 13.5487) |
| Republic of Cyprus | 94.1663 (134.4123, 54.7128) | 123.0835 (174.0945, 81.4073) | 8.2647 (11.9054, 5.4758) | 1718.3857 (2440.6116, 1038.8985) | 1978.8063 (2711.7122, 1299.7997) | 115.8306 (160.0805, 77.4461) |
| Kingdom of Denmark | 720.4589 (1098.3341, 355.2655) | 243.5163 (360.4212, 141.8026) | 1.8178 (2.6834, 1.0543) | 13811.9691 (20709.1819, 7232.8412) | 3916.3654 (5870.8228, 2271.4354) | 33.2313 (49.7272, 19.4663) |
| Republic of Finland | 777.3233 (1139.1566, 421.8087) | 1020.1394 (1386.7224, 671.1011) | 6.3566 (8.4994, 4.3559) | 16528.9093 (23980.0209, 9144.5121) | 14354.3278 (19244.6948, 10073.6747) | 106.3991 (141.8723, 73.8759) |
| French Republic | 4400.4599 (6224.5479, 2901.6904) | 5082.4908 (7011.0175, 3578.2561) | 2.5255 (3.4281, 1.7809) | 74720.7334 (104746.3985, 47528.2083) | 74139.0910 (100139.1223, 52822.1608) | 46.8432 (63.4346, 32.4624) |
| Federal Republic of Germany | 14221.9276 (19995.3453, 8890.7600) | 13546.0744 (18249.4736, 9084.4541) | 5.5100 (7.3919, 3.7085) | 263243.1178 (368295.8930, 160256.8374) | 187976.0296 (251000.2607, 129386.4254) | 88.6254 (118.2055, 61.2501) |
| Hellenic Republic | 46.4276 (128.2282, 4.8705) | 632.5712 (1166.3712, 268.8170) | 1.7670 (3.2321, 0.7748) | 485.3569 (1409.1540, 50.9244) | 7397.3877 (13741.4824, 3272.7593) | 24.3404 (45.5890, 10.5010) |
| Republic of Iceland | 26.6683 (39.0453, 13.8369) | 23.4160 (33.3223, 14.4473) | 3.3903 (4.8403, 2.0787) | 527.3176 (764.0978, 284.1270) | 362.1766 (511.6847, 217.7555) | 60.7191 (85.2577, 36.7067) |
| Ireland | 382.1212 (572.2515, 198.6426) | 177.1975 (264.1453, 101.6556) | 2.1047 (3.1301, 1.2019) | 7958.3544 (11785.3538, 4239.8420) | 2981.4186 (4470.4952, 1640.0159) | 37.9112 (56.6375, 20.7482) |
| State of Israel | 98.9945 (182.1530, 43.2653) | 44.3411 (94.9261, 11.7586) | 0.2945 (0.6254, 0.0830) | 1702.9152 (3233.8387, 724.0274) | 536.9503 (1143.4738, 155.3735) | 3.8169 (8.1819, 1.1102) |
| Republic of Italy | 3351.7389 (4983.8514, 1986.4431) | 12460.9844 (17414.3845, 8029.0464) | 5.8526 (8.0983, 3.8885) | 52935.0449 (79219.0651, 30295.9334) | 149889.4213 (206735.6055, 101331.0933) | 81.5051 (110.9765, 55.9213) |
| Grand Duchy of Luxembourg | 41.7722 (60.6740, 24.0718) | 37.5139 (52.9288, 24.9572) | 2.9767 (4.2093, 1.9719) | 811.3793 (1171.7882, 465.7119) | 545.1648 (765.2469, 361.8712) | 47.5128 (67.3993, 31.4236) |
| Republic of Malta | 30.7729 (45.6131, 17.1579) | 25.6325 (39.6937, 13.6480) | 2.2163 (3.4549, 1.1757) | 614.6377 (917.0714, 339.8580) | 348.1339 (544.4938, 177.0611) | 33.7838 (53.1739, 17.1457) |
| Kingdom of the Netherlands | 1306.5244 (1924.8832, 698.7253) | 974.0267 (1347.9298, 610.7280) | 2.4596 (3.4033, 1.5701) | 27236.8211 (39695.4464, 14731.4914) | 15135.4527 (20973.6264, 9464.3848) | 42.6478 (59.0778, 26.7356) |
| Kingdom of Norway | 613.3873 (912.4510, 309.1731) | 285.3805 (413.6333, 176.7156) | 2.3787 (3.4216, 1.4467) | 11923.0031 (17429.4527, 6224.3343) | 4522.6515 (6559.7093, 2700.1905) | 43.4283 (62.8083, 25.7691) |
| Portuguese Republic | 577.0685 (896.6792, 321.8799) | 737.7618 (1153.4125, 424.0619) | 2.1994 (3.4396, 1.2765) | 10038.7655 (15603.8609, 5487.4665) | 9197.0769 (14339.9978, 5365.2608) | 31.5979 (49.1676, 18.4310) |
| Kingdom of Spain | 1193.2566 (1945.6822, 597.4161) | 3766.2013 (5397.9879, 2383.5275) | 2.6946 (3.7975, 1.7468) | 19334.3938 (32770.8654, 9329.3490) | 49047.3721 (69107.8975, 32525.2611) | 42.3331 (60.3045, 28.3551) |
| Kingdom of Sweden | 1297.8292 (1972.3394, 627.1610) | 1117.6084 (1508.9119, 757.3314) | 3.9615 (5.3914, 2.6791) | 23575.6538 (35175.3315, 12123.8738) | 15553.0302 (21201.9428, 10466.4408) | 64.3376 (88.7550, 42.8214) |
| Swiss Confederation | 961.9848 (1338.2422, 624.0762) | 1305.2453 (1763.5274, 878.1293) | 5.0269 (6.7205, 3.4145) | 15956.5211 (22261.4366, 10184.6700) | 15911.8649 (21088.6268, 10916.7044) | 71.4419 (94.3285, 50.2300) |
| United Kingdom of Great Britain and Northern Ireland | 7801.1595 (11534.3479, 4075.3427) | 4280.4971 (5999.9680, 2764.4749) | 2.9851 (4.1624, 1.9529) | 158670.5456 (232073.3711, 85300.9859) | 76231.0189 (105969.6892, 49301.9110) | 61.9197 (85.7620, 39.7833) |
| Argentine Republic | 4871.7387 (6211.7159, 3587.7194) | 4888.5951 (6319.6963, 3709.9302) | 8.3894 (10.8384, 6.3838) | 102885.4731 (131289.2807, 74363.7473) | 82359.3221 (104673.4301, 63426.1939) | 145.8354 (185.5819, 112.7322) |
| Republic of Chile | 723.0245 (983.4987, 491.9740) | 979.3552 (1332.1307, 673.9510) | 3.7350 (5.1007, 2.5893) | 15509.7466 (20942.0744, 10650.4642) | 17729.9100 (24053.4560, 12470.6899) | 69.7294 (94.4576, 49.4275) |
| Eastern Republic of Uruguay | 550.6694 (783.1107, 315.0529) | 543.0268 (687.9791, 396.3135) | 8.2923 (10.4763, 6.0355) | 12140.7515 (17349.5830, 6684.4432) | 8874.3069 (11289.6641, 6335.3874) | 156.5279 (199.7827, 110.1531) |
| Canada | 1720.3170 (2647.8058, 867.1893) | 1957.3787 (2837.4951, 1150.6001) | 2.5376 (3.6485, 1.5270) | 35528.6141 (53416.8244, 18464.5841) | 38194.0947 (54000.0234, 23805.8277) | 57.6525 (80.1394, 37.4994) |
| United States of America | 22551.9856 (33356.8993, 13158.2171) | 38726.7281 (50967.5082, 27488.4333) | 6.5141 (8.5306, 4.6877) | 475270.0607 (684105.3071, 292488.2426) | 802649.8961 (1044005.8421, 587133.4329) | 154.1755 (198.7569, 115.2893) |
| Antigua and Barbuda | 13.2867 (16.8952, 10.0729) | 21.7800 (26.9796, 17.1451) | 23.9074 (30.1543, 18.6414) | 258.3335 (325.7742, 199.2897) | 445.9965 (539.3699, 356.0213) | 439.4629 (538.3478, 349.7362) |
| Commonwealth of the Bahamas | 29.4948 (38.7748, 21.2044) | 92.3656 (126.5789, 68.0703) | 25.3200 (34.8203, 18.5837) | 749.4550 (976.3995, 547.7601) | 2230.4237 (3066.5769, 1634.7994) | 545.8065 (754.0567, 402.1745) |
| Barbados | 44.8971 (58.4079, 33.0680) | 48.3585 (64.9568, 33.5819) | 9.4647 (12.6840, 6.6178) | 849.1451 (1091.4062, 622.0663) | 924.9913 (1253.1110, 653.2472) | 186.1625 (250.9333, 131.8159) |
| Belize | 16.1404 (21.4515, 11.1423) | 44.9764 (56.6973, 34.4162) | 16.3516 (20.5248, 12.4744) | 379.3279 (503.5829, 248.8411) | 1105.2744 (1375.5690, 832.7005) | 354.8199 (443.4254, 269.0663) |
| Republic of Cuba | 1093.3510 (1563.6586, 655.1563) | 1819.0228 (2434.3035, 1239.1009) | 8.6803 (11.6746, 5.9039) | 24787.3554 (34824.4608, 15271.6563) | 32983.5975 (44732.8976, 22336.7286) | 168.2435 (226.8115, 114.0804) |
| Commonwealth of Dominica | 17.6419 (22.3952, 13.3432) | 18.4623 (24.1062, 13.4505) | 24.2647 (31.8080, 17.6172) | 331.3531 (410.5395, 257.9882) | 362.5697 (470.0477, 266.6512) | 451.0066 (582.5769, 334.3785) |
| Dominican Republic | 640.8333 (870.8787, 438.8489) | 1397.1934 (1946.6356, 906.1849) | 14.2785 (19.9208, 9.2863) | 16743.9372 (22979.6765, 11181.0389) | 32814.6297 (45211.7385, 21550.5579) | 321.6784 (444.0619, 211.0074) |
| Grenada | 25.6884 (35.3101, 15.7410) | 23.9935 (31.2148, 16.8095) | 24.2051 (31.5710, 16.9729) | 582.3692 (798.7940, 338.2757) | 557.9753 (729.0738, 385.2412) | 502.1883 (657.9529, 350.6436) |
| Republic of Guyana | 268.1085 (355.5127, 171.3712) | 199.6103 (270.8511, 143.4614) | 35.7977 (47.3455, 25.8727) | 7155.6591 (9638.9499, 4356.5879) | 5091.6271 (6969.1508, 3605.0234) | 786.2600 (1071.5496, 564.1782) |
| Republic of Haiti | 1662.5828 (2470.1573, 800.7895) | 3106.2472 (4796.3334, 1551.2220) | 48.1671 (73.8897, 25.2316) | 47986.5118 (71330.5802, 21914.9985) | 91101.5339 (140999.3088, 41684.6584) | 1138.8779 (1751.3936, 568.7263) |
| Jamaica | 475.9837 (572.8455, 382.0939) | 559.1577 (732.8699, 407.9192) | 16.4439 (21.8392, 11.8766) | 8879.4553 (10659.0166, 7159.3633) | 10712.9501 (14146.7088, 7824.2356) | 335.9047 (441.5733, 242.4642) |
| Saint Lucia | 29.2969 (39.6896, 19.0563) | 43.3249 (56.6766, 31.6028) | 18.9527 (24.8194, 13.7199) | 665.0870 (905.4008, 412.2179) | 878.9180 (1175.6906, 638.7356) | 373.1438 (497.4520, 271.1787) |
| Saint Vincent and the Grenadines | 29.6973 (38.8527, 20.0774) | 43.6155 (53.6993, 33.8313) | 35.0524 (42.9873, 27.2507) | 661.5334 (873.8084, 432.8447) | 888.5859 (1107.4483, 697.0437) | 661.5320 (814.8014, 521.0173) |
| Republic of Suriname | 61.6990 (79.7166, 42.8143) | 105.2228 (146.8662, 70.3479) | 17.4468 (24.5276, 11.6593) | 1508.7213 (1958.4709, 1014.1798) | 2496.7415 (3446.5413, 1709.4753) | 393.0320 (542.6403, 271.0039) |
| Republic of Trinidad and Tobago | 260.9179 (340.5872, 178.1932) | 272.6772 (379.6158, 178.2742) | 14.7290 (20.3941, 9.5810) | 6227.6513 (8149.9842, 4150.8847) | 6308.8474 (8809.6914, 4045.6307) | 338.9309 (470.1511, 218.7342) |
| Plurinational State of Bolivia | 619.0759 (891.2409, 370.3228) | 1131.0317 (1643.5230, 682.3450) | 14.6559 (21.2190, 9.0259) | 15519.9233 (22633.0085, 8917.4057) | 26296.9389 (38438.0774, 15093.5368) | 295.2483 (429.9476, 177.6607) |
| Republic of Ecuador | 1363.2954 (1742.3295, 945.2681) | 2245.6562 (3093.0659, 1490.9066) | 15.5499 (21.3880, 10.5644) | 31934.8471 (42049.7971, 20194.8770) | 41657.6343 (57544.6366, 27012.3542) | 267.4652 (367.7534, 176.4165) |
| Republic of Peru | 1258.5065 (1674.3497, 850.2345) | 1520.6053 (2165.7415, 965.9284) | 4.4872 (6.4254, 2.8593) | 29768.8759 (41050.5777, 17557.1273) | 34486.5870 (48244.0015, 21556.2043) | 99.9164 (140.0417, 62.7538) |
| Republic of Colombia | 2888.7091 (3701.4649, 2119.1646) | 3345.9198 (4619.7198, 2228.3095) | 5.8707 (8.1462, 3.9071) | 66888.7377 (85246.7183, 47802.9052) | 66935.7960 (92923.6909, 42477.6213) | 120.1307 (166.8791, 76.1842) |
| Republic of Costa Rica | 240.7776 (335.8054, 142.3658) | 358.1154 (476.2522, 241.7198) | 6.2744 (8.3835, 4.2325) | 5625.4840 (7953.1112, 3114.5356) | 7181.0440 (9666.4876, 4665.9216) | 129.1808 (174.1486, 83.7232) |
| Republic of El Salvador | 393.2118 (566.8568, 202.2939) | 417.1390 (601.2046, 244.1661) | 6.1906 (8.9593, 3.5805) | 10287.6883 (14932.4706, 4780.4587) | 8548.2618 (12435.7716, 4952.1312) | 134.9226 (197.2980, 77.8741) |
| Republic of Guatemala | 350.2650 (484.1230, 205.5607) | 605.7680 (853.8997, 361.4173) | 6.6095 (9.3506, 4.0323) | 9721.0093 (13590.5206, 5286.3172) | 13350.2044 (18924.4717, 7804.5739) | 124.3299 (175.5420, 74.2512) |
| Republic of Honduras | 412.4069 (582.8683, 250.9490) | 1100.9416 (1475.2349, 747.1315) | 21.4192 (28.7198, 14.6751) | 11302.4254 (15938.6716, 6389.9479) | 25124.9043 (33488.7976, 17020.8881) | 412.9320 (549.0368, 283.6061) |
| United Mexican States | 4020.1745 (5242.6756, 2786.0163) | 8596.6625 (11997.2837, 5560.1996) | 7.5191 (10.4113, 4.8899) | 88824.8869 (117460.7491, 59969.4495) | 184514.2472 (259884.8836, 116687.8473) | 148.1383 (208.3683, 94.4607) |
| Republic of Nicaragua | 288.0471 (404.0613, 159.6816) | 610.0692 (862.4188, 340.3630) | 13.9907 (19.5361, 7.9396) | 7663.3615 (10825.5810, 3796.0327) | 14918.7355 (21993.9288, 7955.9745) | 299.4375 (434.0394, 164.2663) |
| Republic of Panama | 152.1099 (222.5485, 77.0898) | 308.0200 (428.9932, 186.1702) | 6.7264 (9.4238, 4.0638) | 3655.2179 (5393.8010, 1744.6012) | 6439.6067 (9174.6831, 3757.4791) | 144.1925 (206.0965, 84.2164) |
| Bolivarian Republic of Venezuela | 1949.1117 (2588.3658, 1310.8024) | 3827.5726 (5340.3465, 2427.6898) | 13.4650 (18.6077, 8.6035) | 50213.0561 (67124.7609, 32497.9333) | 84451.9446 (119222.0438, 52363.6001) | 283.1633 (396.9270, 175.3604) |
| Federative Republic of Brazil | 17411.6108 (23129.3993, 11233.5738) | 20839.2669 (26609.0715, 15064.9436) | 8.5416 (10.8986, 6.2254) | 476863.0933 (639744.3677, 299408.7054) | 477751.0296 (620378.1427, 338106.1841) | 190.0017 (246.6605, 135.0236) |
| Republic of Paraguay | 337.6643 (456.7109, 216.7669) | 713.9494 (972.3415, 484.1246) | 13.0510 (17.8589, 8.9386) | 8054.5705 (11010.4771, 4920.3326) | 15823.4161 (21767.3025, 10519.8794) | 270.2712 (371.1793, 181.3393) |
| People's Democratic Republic of Algeria | 2932.6251 (4142.7353, 1959.8001) | 5323.7463 (7555.6484, 3341.8157) | 21.2276 (30.5228, 13.4421) | 73217.2581 (101467.8665, 48333.7403) | 112607.4065 (163094.1399, 71035.8678) | 353.9897 (499.7726, 226.3993) |
| Kingdom of Bahrain | 18.1057 (27.4654, 10.1582) | 26.9948 (43.4253, 13.5478) | 6.2115 (9.9595, 3.3298) | 509.0639 (762.8342, 289.8371) | 717.8230 (1189.1818, 347.6929) | 98.3093 (155.9617, 51.5559) |
| Arab Republic of Egypt | 3408.0067 (5920.4883, 1681.9919) | 574.0672 (1582.0942, 79.1791) | 2.4192 (6.3307, 0.3734) | 77220.4253 (139260.6877, 36166.3577) | 9791.4345 (25686.8229, 1258.6876) | 29.9313 (78.2635, 4.4103) |
| Islamic Republic of Iran | 4471.9067 (6022.4274, 3179.5215) | 4723.7597 (7141.4346, 2898.0110) | 7.4915 (11.1736, 4.6361) | 113189.6965 (153065.9837, 80383.6487) | 80922.3186 (125660.6356, 48318.0901) | 114.0845 (175.5396, 68.7713) |
| Republic of Iraq | 933.2141 (1438.2606, 545.5558) | 1712.4741 (2709.2115, 942.1693) | 10.0202 (15.2918, 5.7471) | 21670.0827 (33655.9453, 12381.3425) | 39627.1380 (64273.1744, 20963.8346) | 178.8511 (283.6591, 98.2102) |
| Hashemite Kingdom of Jordan | 269.0481 (366.1443, 192.0433) | 742.8909 (1013.3883, 485.9547) | 14.0855 (19.6310, 9.1012) | 6888.9012 (9487.3626, 4833.2880) | 17937.2413 (24983.5208, 12036.9740) | 256.6538 (348.9021, 168.0588) |
| State of Kuwait | 52.4315 (78.3526, 31.2743) | 66.9346 (120.0280, 28.6448) | 2.7939 (4.9952, 1.2521) | 1483.4642 (2269.8782, 837.3448) | 1693.1430 (3107.8815, 672.7777) | 51.7747 (93.6433, 21.4677) |
| Lebanese Republic | 25.7665 (60.1219, 4.3583) | 82.8069 (173.7718, 21.3609) | 1.1770 (2.4661, 0.3078) | 294.5509 (741.3805, 46.9950) | 1050.1120 (2231.5002, 259.4942) | 15.3798 (32.5914, 3.7868) |
| State of Libya | 175.2261 (272.9225, 100.6774) | 660.8390 (1036.1135, 393.4018) | 14.7611 (23.0403, 8.9587) | 4166.3641 (6521.0646, 2367.0367) | 16580.5764 (26189.1909, 9961.1007) | 300.4398 (474.0573, 180.6731) |
| Kingdom of Morocco | 3471.7752 (4845.0978, 2262.5882) | 4049.3633 (6362.1311, 2284.5376) | 14.2466 (22.3900, 8.1034) | 86230.4593 (119419.5927, 55353.9878) | 87490.7722 (141008.6043, 48672.3273) | 267.7846 (424.2895, 150.4821) |
| Palestine | 438.6498 (609.6278, 279.7131) | 364.9702 (487.0820, 252.4636) | 22.5392 (30.3319, 15.7113) | 9867.7623 (13890.7302, 6003.2099) | 7587.8248 (10171.2417, 5154.8471) | 355.9242 (472.2816, 246.8945) |
| Sultanate of Oman | 167.7772 (245.4714, 108.2673) | 222.6539 (310.8162, 147.0154) | 16.1735 (22.8512, 10.5345) | 4172.9084 (6097.1719, 2588.6176) | 5854.8699 (8181.9624, 3843.5175) | 291.7261 (407.2181, 193.7213) |
| State of Qatar | 4.1721 (8.1013, 1.6603) | 3.1634 (7.6036, 0.5883) | 0.7821 (1.8125, 0.1301) | 123.5946 (245.9018, 42.6295) | 102.9002 (275.8070, 14.1760) | 11.7183 (27.6405, 2.3342) |
| Kingdom of Saudi Arabia | 1364.5964 (2007.1954, 847.9580) | 3503.2622 (5233.6410, 2086.8090) | 21.9678 (31.8872, 13.2576) | 33959.7006 (50588.3845, 19979.6782) | 116288.4941 (176341.5800, 67661.0554) | 467.0830 (679.9748, 285.5626) |
| Syrian Arab Republic | 1030.0645 (1457.3471, 650.2389) | 2459.0157 (3624.1678, 1494.9572) | 25.2512 (36.2911, 16.2509) | 24333.4733 (35663.8695, 14763.1506) | 58296.6481 (87080.0690, 35255.1898) | 476.7858 (697.3579, 295.7992) |
| Republic of Tunisia | 847.3078 (1193.7667, 572.4316) | 1135.9952 (1970.5615, 545.4958) | 10.4258 (18.0388, 5.0482) | 18965.2429 (26755.4483, 13037.8260) | 19087.1002 (33958.5991, 8655.2384) | 160.1233 (278.7263, 75.6883) |
| Republic of Turkey | 536.6290 (1267.6821, 127.6770) | 346.7355 (1158.8246, 19.6541) | 0.4782 (1.6230, 0.0261) | 8920.8366 (21475.9426, 2060.0092) | 4047.0929 (12781.6952, 312.7180) | 5.3864 (17.5437, 0.3819) |
| United Arab Emirates | 4.4393 (10.2502, 1.2176) | 239.8815 (347.8016, 148.8286) | 13.5153 (19.0503, 8.6650) | 99.8918 (262.6020, 19.4935) | 8597.2906 (12450.6014, 5405.0027) | 236.0215 (334.4711, 154.5078) |
| Republic of Yemen | 2427.2988 (3422.1629, 1448.8592) | 5763.5948 (8618.0175, 3292.8964) | 50.5175 (75.2502, 30.2933) | 65510.9526 (94637.4404, 37896.7887) | 150073.3104 (224215.8538, 82163.8960) | 1036.5696 (1537.8050, 588.4762) |
| Islamic Republic of Afghanistan | 6958.2701 (10023.3739, 3918.6124) | 7196.6306 (10314.1428, 4198.6748) | 82.2048 (115.8858, 49.8655) | 186609.8044 (268796.9972, 102382.5172) | 218938.4075 (317120.2005, 119793.1584) | 1891.7649 (2688.4223, 1118.1696) |
| People's Republic of Bangladesh | 21284.4716 (31451.1048, 9804.9483) | 29039.7193 (45955.9395, 14208.7992) | 24.4834 (38.8166, 12.5451) | 623287.5677 (913229.6485, 254543.4675) | 707211.6835 (1092380.9537, 319434.6543) | 517.3056 (798.4614, 246.7842) |
| Kingdom of Bhutan | 48.8589 (73.1692, 26.2880) | 78.7760 (111.5436, 50.9354) | 14.3560 (20.2263, 9.3965) | 1411.5582 (2100.4795, 695.5564) | 1712.5513 (2400.0973, 1069.1737) | 282.9187 (396.2093, 179.3403) |
| Republic of India | 64983.1942 (91455.9443, 41135.8357) | 140780.4748 (187935.6148, 93832.9585) | 13.1821 (17.6989, 8.9256) | 1919623.6888 (2670328.2088, 1180355.2027) | 3625548.1066 (4904253.9146, 2341430.1808) | 299.0641 (401.5546, 197.0807) |
| Federal Democratic Republic of Nepal | 2224.7207 (3240.7022, 1181.1413) | 3603.3084 (5002.0640, 2471.3216) | 19.4917 (27.2339, 13.3829) | 62782.4926 (92790.0021, 31008.3488) | 81106.1021 (112700.4885, 53672.8922) | 370.7206 (510.3383, 251.5214) |
| Islamic Republic of Pakistan | 13595.3006 (19800.9034, 7470.6279) | 25092.9981 (37786.9206, 14623.8462) | 23.9770 (35.2739, 14.6173) | 369211.1446 (542468.9282, 187168.2899) | 718741.5157 (1091802.1432, 391351.6839) | 539.6388 (807.3151, 309.1798) |
| Republic of Angola | 2010.0238 (2835.4652, 1196.1098) | 2595.4030 (3593.7433, 1674.0766) | 31.4444 (43.2461, 20.0110) | 59216.1354 (84941.2852, 33195.7602) | 69201.7680 (98467.2280, 44208.4688) | 615.5115 (843.4407, 399.5431) |
| Central African Republic | 719.7408 (1045.2460, 372.5296) | 1191.4812 (1811.7740, 614.8213) | 66.0110 (98.7369, 37.3427) | 21387.4760 (31435.9095, 11041.2977) | 36698.3720 (56252.8842, 18061.0571) | 1521.7000 (2306.0602, 818.0117) |
| Republic of the Congo | 666.9691 (958.4503, 377.5323) | 1160.2225 (1755.6749, 665.5842) | 53.0974 (76.0899, 32.1539) | 18870.2261 (27493.0284, 10142.8980) | 33308.8945 (50420.1317, 18607.3178) | 1163.2095 (1728.8682, 673.7579) |
| Democratic Republic of the Congo | 6540.6990 (9765.7508, 3659.9571) | 15309.8082 (22122.2811, 8247.2967) | 53.4157 (75.5429, 29.9932) | 186926.3202 (280753.2961, 98914.9709) | 420121.1100 (611982.7958, 217264.7693) | 1136.3580 (1634.0099, 619.4359) |
| Republic of Equatorial Guinea | 119.9681 (174.8984, 63.4374) | 124.3729 (198.2350, 69.7378) | 30.9000 (49.1993, 18.3497) | 3386.2539 (5060.0929, 1692.1710) | 3392.2728 (5441.5100, 1855.6156) | 640.1119 (1014.9607, 366.6995) |
| Gabonese Republic | 257.3001 (356.5119, 167.5101) | 312.2021 (446.1879, 199.5460) | 38.2746 (55.5825, 25.1042) | 6127.0919 (8584.8425, 3863.9359) | 7728.7514 (11206.6593, 4742.3048) | 765.7753 (1089.4992, 492.2050) |
| Republic of Burundi | 1151.2987 (1688.7703, 533.8127) | 1283.8309 (1868.4746, 653.0531) | 31.8111 (45.4744, 17.2586) | 32184.1327 (47598.4372, 14083.4837) | 37589.3789 (54729.1804, 18197.4648) | 708.7354 (1021.4627, 366.9179) |
| Union of the Comoros | 93.4444 (134.9178, 49.9914) | 153.9000 (227.1383, 79.8644) | 36.5445 (53.9966, 20.0592) | 2652.7159 (3942.0216, 1339.5427) | 4011.5102 (5818.8548, 1979.5514) | 803.2004 (1175.0207, 418.6755) |
| Republic of Djibouti | 40.4680 (56.7463, 24.8842) | 105.4681 (160.2852, 65.0330) | 23.5924 (35.8729, 14.8414) | 1195.4715 (1681.4426, 718.9269) | 3015.4543 (4563.5000, 1903.3164) | 472.1102 (707.9639, 301.0754) |
| State of Eritrea | 690.5811 (980.2643, 394.6818) | 1068.0104 (1568.3209, 606.9541) | 51.2323 (71.8403, 31.1219) | 22158.7177 (32211.8810, 12066.4877) | 31449.1477 (47279.6778, 17139.2231) | 1086.7439 (1558.1782, 638.0592) |
| Federal Democratic Republic of Ethiopia | 11126.5587 (14946.3828, 6940.9059) | 10379.8486 (14027.9907, 6875.7040) | 28.7662 (38.2818, 19.5106) | 318553.0967 (428089.6075, 189222.9107) | 261021.6417 (355417.3962, 162742.0294) | 592.0238 (803.7773, 386.7287) |
| Republic of Kenya | 1337.7476 (1784.4457, 955.1607) | 3095.2041 (4200.7717, 2109.4519) | 18.9533 (26.1318, 12.6615) | 33845.3067 (44881.0772, 23070.5582) | 78052.6799 (104324.1735, 53872.1282) | 360.0982 (484.5730, 247.8370) |
| Republic of Madagascar | 3268.8732 (4423.4920, 1970.8191) | 5909.5170 (8629.7737, 3166.8155) | 62.7669 (88.4771, 37.6168) | 91872.9020 (126383.0645, 52675.8960) | 185438.4274 (273850.9198, 97136.7165) | 1447.0551 (2084.2985, 793.2806) |
| Republic of Malawi | 1081.8165 (1542.6905, 591.8025) | 1992.9494 (2895.3719, 1086.1762) | 32.2091 (46.6563, 18.9131) | 30617.5668 (44290.9234, 15982.6638) | 57049.3386 (84078.3212, 28946.5486) | 716.3786 (1030.3212, 390.9105) |
| Republic of Mauritius | 212.1827 (270.0563, 154.2143) | 229.3396 (294.5762, 173.5719) | 13.6671 (17.6671, 10.3825) | 5778.2949 (7400.3258, 4086.5870) | 5162.0389 (6619.5356, 3920.7923) | 298.7215 (380.6314, 225.8078) |
| Republic of Mozambique | 2627.9237 (3686.7494, 1437.3928) | 4429.9093 (6564.3426, 2149.5341) | 50.3168 (73.0990, 26.4666) | 70808.0802 (99745.7079, 35634.4168) | 125290.5988 (189726.6030, 58805.7676) | 1077.9653 (1588.5423, 527.6068) |
| Republic of Rwanda | 2010.6045 (2817.2457, 1011.0844) | 1376.3899 (1989.9999, 729.2004) | 30.0282 (44.2225, 16.1316) | 58839.0140 (83624.6568, 27797.2165) | 34845.5281 (50074.2617, 18108.6924) | 579.6085 (829.4134, 314.1076) |
| Republic of Seychelles | 28.2658 (36.5064, 21.3939) | 29.3249 (37.5195, 22.0850) | 28.7779 (36.6601, 21.3622) | 634.0670 (810.1916, 462.5481) | 678.4743 (872.4311, 498.4451) | 593.9074 (756.8673, 440.0384) |
| Federal Republic of Somalia | 1451.7028 (2149.5032, 749.2477) | 2472.3142 (3745.3292, 1254.5053) | 50.0537 (72.3587, 26.2390) | 45584.6331 (68396.5418, 21632.1044) | 77879.7642 (121577.6586, 38702.7729) | 1143.4600 (1694.8622, 594.6856) |
| United Republic of Tanzania | 3235.4026 (4385.4719, 1944.2334) | 5172.4833 (7584.3667, 2951.0931) | 25.8032 (38.5261, 14.7953) | 84729.1469 (116488.1527, 51052.0258) | 125347.6979 (178514.4352, 72076.6397) | 500.2797 (726.3570, 293.3895) |
| Republic of Uganda | 1960.9527 (3098.4303, 920.5124) | 2930.5539 (4418.8688, 1472.0316) | 24.1593 (36.3916, 13.2062) | 54345.9987 (86659.0604, 24109.8357) | 82217.3363 (123403.4795, 39526.8575) | 525.7319 (787.8553, 266.2413) |
| Republic of Zambia | 1363.6243 (1863.8117, 903.3535) | 2724.6767 (3879.0925, 1623.2477) | 52.1337 (72.3943, 32.5533) | 35851.5053 (48581.9641, 22481.5256) | 72955.8024 (108022.2125, 40613.2714) | 1044.0871 (1485.4712, 629.6523) |
| Republic of Botswana | 195.9821 (286.4020, 114.2811) | 293.7124 (404.1591, 201.3688) | 25.9342 (35.4362, 18.1039) | 5338.9677 (7913.5675, 3038.4442) | 7460.4277 (10486.0644, 4896.3784) | 518.0195 (711.9359, 353.2485) |
| Kingdom of Lesotho | 301.1505 (417.1979, 191.5970) | 522.6945 (748.2142, 302.4154) | 57.4932 (79.1968, 34.8736) | 7038.9523 (9726.2422, 4382.7135) | 13855.0116 (19923.2713, 7796.5884) | 1274.5603 (1813.1371, 743.7533) |
| Republic of Namibia | 290.6323 (408.4353, 172.7314) | 401.9892 (565.7634, 257.1065) | 37.9422 (53.1068, 24.6355) | 7842.9495 (11101.3714, 4519.9533) | 9802.5621 (13999.7670, 6175.4085) | 743.9604 (1049.1377, 476.0325) |
| Republic of South Africa | 3580.5828 (4720.7215, 2634.5212) | 8191.2823 (10591.3005, 6116.4643) | 21.0818 (27.4429, 15.4710) | 99819.6214 (126840.3086, 72291.2551) | 201282.9721 (257913.2671, 150440.7580) | 435.2087 (562.5826, 324.3346) |
| Kingdom of Eswatini | 115.7147 (161.8501, 72.1158) | 215.9021 (325.1075, 126.0989) | 45.7046 (67.3014, 27.5489) | 3116.9948 (4355.9364, 1809.8828) | 6278.0702 (9455.4679, 3515.2678) | 1047.2350 (1567.5424, 617.5610) |
| Republic of Zimbabwe | 1110.5487 (1568.2364, 665.0832) | 2664.8625 (3993.0311, 1438.9762) | 44.1091 (65.2156, 25.2132) | 29184.6715 (41467.8167, 16601.9291) | 79303.7106 (118581.1724, 41369.4506) | 1052.0785 (1567.9942, 572.3159) |
| Republic of Benin | 328.2766 (455.5129, 218.1966) | 637.1485 (898.0287, 429.2144) | 14.3784 (20.2872, 9.6128) | 8399.2935 (11483.5925, 5501.2989) | 17184.4237 (24075.1273, 11676.7716) | 313.9009 (433.0830, 214.0481) |
| Burkina Faso | 1202.2338 (1667.5682, 715.7959) | 2770.1256 (3926.4276, 1585.3054) | 33.8188 (47.7092, 20.0414) | 33160.6144 (45708.4032, 18966.2827) | 74236.6971 (105633.5615, 41143.0280) | 764.3822 (1077.1068, 440.0852) |
| Republic of Cameroon | 978.6594 (1320.7692, 618.2634) | 1585.6859 (2428.5036, 896.0346) | 15.1166 (23.2061, 8.6553) | 26601.4524 (35168.2476, 17182.7095) | 45300.2346 (69099.9892, 25660.4593) | 331.3954 (504.3642, 191.2949) |
| Republic of Cabo Verde | 41.2678 (57.5217, 27.6398) | 43.9668 (60.6675, 28.8469) | 10.3394 (14.2760, 6.7873) | 970.8769 (1352.8232, 635.7605) | 934.0424 (1290.7531, 621.3906) | 205.0982 (284.8332, 137.0543) |
| Republic of Chad | 1040.2479 (1506.2260, 579.8930) | 2229.2070 (3368.0315, 1180.7116) | 42.3562 (63.1570, 23.7697) | 27959.5447 (40283.9774, 14630.6417) | 65646.6088 (99168.0358, 33410.1411) | 1026.2441 (1543.2926, 544.9954) |
| Republic of C么te d'Ivoire | 769.2040 (1061.8217, 466.7984) | 2094.8385 (3063.0299, 1228.0579) | 21.5454 (31.2287, 13.0746) | 23871.7483 (33018.7360, 14499.8327) | 63434.0984 (93238.8214, 36416.9711) | 498.5168 (723.5569, 295.1709) |
| Republic of the Gambia | 109.4195 (161.6556, 56.3313) | 316.8217 (468.4744, 175.0433) | 35.3621 (52.3721, 20.2325) | 3283.2084 (4869.2768, 1612.5180) | 8959.1105 (13251.9199, 4735.4352) | 831.0029 (1233.0176, 447.1868) |
| Republic of Ghana | 1629.0071 (2243.1650, 970.7406) | 2887.9260 (4094.5396, 1858.8050) | 19.3555 (27.3602, 12.6235) | 49689.0939 (68859.1031, 28957.9197) | 86194.3583 (121467.1873, 55660.0112) | 461.4697 (651.3366, 299.8584) |
| Republic of Guinea | 674.5127 (959.6292, 436.9562) | 1292.4637 (1880.3904, 800.1383) | 25.4060 (36.4177, 16.0685) | 17251.9753 (24473.8856, 11245.1109) | 35086.8005 (51789.3044, 21758.0950) | 583.1105 (855.2481, 365.1354) |
| Republic of Guinea-Bissau | 241.7840 (353.0846, 117.1197) | 372.9864 (558.3885, 182.1582) | 55.7433 (82.5199, 29.1074) | 7474.9122 (10945.9164, 3427.2466) | 12119.9228 (18244.2861, 5822.9288) | 1389.2769 (2061.4485, 691.0565) |
| Republic of Liberia | 337.7987 (470.9752, 192.9913) | 662.1618 (1006.5664, 340.4890) | 34.1627 (51.4573, 18.8477) | 9178.7526 (12965.6819, 5097.4157) | 20236.5910 (31032.7193, 9962.2102) | 808.5191 (1220.0896, 421.1273) |
| Republic of Mali | 1078.4339 (1530.5285, 586.1891) | 1768.0192 (2531.3586, 942.9396) | 21.9455 (31.0464, 11.6548) | 31996.4184 (45656.0425, 16991.2395) | 52874.1262 (76157.4162, 28815.6190) | 538.4853 (765.3943, 289.2762) |
| Islamic Republic of Mauritania | 484.4305 (711.1906, 247.1626) | 580.0196 (864.9345, 337.4840) | 30.0212 (44.9243, 17.7809) | 13193.6750 (19338.5462, 6309.3703) | 15040.4393 (22596.8110, 8363.1248) | 672.4819 (994.8303, 386.6850) |
| Republic of the Niger | 410.6232 (597.1792, 228.4155) | 895.7342 (1374.1764, 475.5013) | 13.3379 (20.2345, 7.2146) | 11917.2016 (17196.1669, 6789.3319) | 24803.1624 (37388.1789, 13564.2858) | 294.2618 (445.8622, 162.0110) |
| Federal Republic of Nigeria | 9102.4468 (12576.6225, 6390.7232) | 9164.9671 (12388.2173, 5612.7068) | 12.1805 (16.3274, 7.8185) | 228514.2388 (317713.8800, 154749.7395) | 245887.4989 (336032.7874, 150186.2720) | 256.8538 (344.7503, 159.4954) |
| Democratic Republic of Sao Tome and Principe | 14.0262 (20.5345, 6.9305) | 18.1495 (27.2105, 9.2098) | 18.0834 (27.0632, 9.5634) | 379.6360 (549.3191, 177.0015) | 538.0235 (824.3201, 263.6760) | 434.8621 (650.8229, 224.2542) |
| Republic of Senegal | 711.8431 (1008.1439, 420.4291) | 1279.0702 (1833.7485, 831.4107) | 18.5730 (26.7387, 12.0871) | 20198.2390 (29020.8753, 11704.8553) | 34755.9047 (49686.4229, 22772.1946) | 423.3460 (599.8434, 279.1753) |
| Republic of Sierra Leone | 426.0422 (627.1858, 233.1924) | 712.9655 (1044.3306, 395.2371) | 20.4946 (30.1709, 11.6846) | 11739.7762 (17279.1416, 6252.0538) | 20863.5143 (30450.2302, 11124.4253) | 491.1944 (716.9749, 269.4603) |
| Togolese Republic | 365.1840 (523.9894, 190.0384) | 1198.5600 (1824.4818, 619.5897) | 34.5578 (52.0871, 18.3336) | 11145.6792 (16349.6398, 5566.2766) | 36996.6725 (56190.5127, 18307.6214) | 846.0120 (1279.4148, 439.4927) |
| American Samoa | 4.3872 (6.0983, 2.4260) | 7.8592 (11.5677, 4.1159) | 17.9132 (26.0704, 9.7352) | 139.1578 (198.9531, 74.4568) | 227.7447 (337.4695, 114.2833) | 458.6643 (671.2070, 233.7320) |
| Bermuda | 5.7750 (8.4980, 3.3973) | 8.4873 (11.3331, 5.8487) | 5.5807 (7.5089, 3.8824) | 122.3935 (178.6197, 73.2311) | 145.8066 (197.5913, 101.6024) | 107.4812 (146.5842, 74.4358) |
| Cook Islands | 8.5486 (10.8461, 6.6113) | 8.3847 (10.6939, 6.2376) | 33.8809 (43.3553, 25.2104) | 217.9215 (282.4281, 168.1926) | 182.5044 (237.5358, 135.4239) | 748.0533 (975.6633, 550.5549) |
| Greenland | 2.8810 (4.1357, 1.7238) | 2.2025 (3.3605, 1.3280) | 3.7892 (5.7417, 2.3156) | 89.8329 (129.5645, 54.9642) | 60.8466 (92.3093, 36.7363) | 89.5906 (132.4146, 54.6421) |
| Guam | 20.7111 (26.2545, 13.7752) | 23.4199 (31.7736, 15.4085) | 11.1507 (15.1961, 7.4168) | 573.9977 (728.2304, 392.0949) | 677.6388 (927.1854, 454.9114) | 338.6576 (463.0645, 228.2361) |
| Principality of Monaco | 1.5193 (2.9333, 0.5819) | 1.0856 (2.3950, 0.3591) | 0.8379 (1.7418, 0.2786) | 23.4958 (45.4628, 9.5569) | 14.0852 (28.8699, 5.0261) | 12.8415 (25.2531, 4.5146) |
| Republic of Nauru | 2.2916 (3.3218, 1.1834) | 2.7582 (4.2457, 1.4051) | 48.5220 (73.1502, 25.7025) | 80.0886 (117.0706, 40.3747) | 95.7169 (149.1396, 47.0717) | 1339.5128 (2057.5502, 682.5940) |
| Republic of Niue | 0.8418 (1.2103, 0.4613) | 0.5310 (0.7853, 0.2953) | 25.7446 (38.0836, 14.2961) | 19.7690 (29.4123, 10.1801) | 13.3797 (20.1913, 7.0790) | 640.9731 (967.1834, 337.9338) |
| Northern Mariana Islands | 2.2857 (3.4237, 1.1678) | 5.4557 (7.7663, 3.0264) | 12.0082 (17.2983, 6.7677) | 85.6543 (128.3099, 42.2509) | 164.0478 (234.1909, 90.1654) | 297.0129 (423.8044, 164.1233) |
| Republic of Palau | 2.6079 (3.9688, 1.1634) | 4.0467 (6.2258, 1.9687) | 20.4129 (31.3573, 10.1408) | 81.9515 (125.3324, 34.9187) | 128.2760 (199.1885, 59.3909) | 547.9799 (839.1283, 265.3825) |
| Puerto Rico | 733.1986 (1008.8266, 433.6679) | 756.4757 (999.1758, 522.1327) | 8.7990 (11.6592, 6.1239) | 16677.4537 (22810.5526, 9732.8310) | 13191.8801 (17366.4587, 9175.7621) | 194.5443 (257.5120, 132.7380) |
| Saint Kitts and Nevis | 10.8927 (14.7642, 6.8734) | 10.3104 (13.4101, 7.7713) | 19.1950 (24.6379, 14.5321) | 238.2628 (322.7750, 147.4995) | 230.8271 (304.0871, 167.9735) | 358.5742 (467.9788, 268.5250) |
| Republic of San Marino | 1.6659 (2.4050, 1.0446) | 2.1915 (3.3449, 1.2403) | 1.9444 (3.0179, 1.1201) | 26.5120 (38.1974, 16.8218) | 29.9048 (45.8527, 17.8365) | 33.5141 (52.1459, 19.6620) |
| Tokelau | 0.6501 (0.9471, 0.3510) | 0.4094 (0.5865, 0.2302) | 28.0089 (40.2321, 15.7716) | 16.3509 (24.0464, 8.2548) | 10.0314 (14.7085, 5.3518) | 695.5720 (1017.1716, 371.9277) |
| Tuvalu | 4.2986 (6.0975, 2.0873) | 4.1850 (6.0356, 2.1913) | 44.0457 (63.0859, 22.8142) | 129.7166 (186.9874, 60.7728) | 121.7822 (175.3658, 61.6084) | 1139.0746 (1642.9710, 588.5627) |
| United States Virgin Islands | 16.0193 (20.9814, 11.4194) | 17.6615 (24.1107, 11.7974) | 10.7577 (14.7612, 7.2538) | 406.1907 (532.6546, 286.1812) | 349.2039 (467.5478, 235.5808) | 221.2747 (296.6054, 150.7154) |
| Republic of South Sudan | 928.6284 (1354.7340, 548.5592) | 889.2574 (1367.0277, 516.6323) | 28.2952 (42.4698, 16.3698) | 23694.9983 (35149.3267, 13393.5234) | 25140.9764 (39273.0748, 14283.4272) | 620.6244 (943.2928, 365.1415) |
| Republic of Sudan | 3161.4831 (4396.7151, 2037.3586) | 4160.7437 (6166.2008, 2556.6350) | 24.7305 (36.9275, 15.3369) | 84206.5811 (116422.8693, 53170.4689) | 110742.1401 (163845.6834, 68026.5737) | 524.3731 (776.8098, 323.5270) |
